# Pharmacophore-based virtual screening and *in silico* investigations of small molecule library for discovery of human hepatic ketohexokinase inhibitors for the treatment of fructose metabolic disorders

**DOI:** 10.3389/fphar.2025.1531512

**Published:** 2025-04-07

**Authors:** Tilal Elsaman, Magdi Awadalla Mohamed, Abozer Y. Elderdery, Abdullah Alsrhani, Badr Alzahrani, Heba Bassiony Ghanem, Jeremy Mills, Musaed Rayzah, Nasser A. N. Alzerwi, Afnan Al-sultan, Bandar Idrees, Fares Rayzah

**Affiliations:** ^1^ Department of Pharmaceutical Chemistry, College of Pharmacy, Jouf University, Sakaka, Saudi Arabia; ^2^ Department of Clinical Laboratory Sciences, College of Applied Medical Sciences, Jouf University, Sakaka, Saudi Arabia; ^3^ School of Medicine, Pharmacy and Biomedical Sciences, Uinversity of Portsmouth, Portsmouth, United Kingdom; ^4^ Department of Surgery, College of Medicine, Majmaah University, Al Majma’ah, Saudi Arabia; ^5^ Department of Surgery, King Saud Medical City, Riyadh, Saudi Arabia; ^6^ Department of Surgery, Prince Sultan Military Medical City, As Sulimaniyah, Saudi Arabia; ^7^ Department of Surgery, Aseer Central Hospital, Abha, Saudi Arabia

**Keywords:** fructose, diabetes, obesity, dyslipidemia, ketohexokinase

## Abstract

**Introduction:**

Excessive fructose consumption is a significant driver of metabolic disorders, including obesity, diabetes, non-alcoholic fatty liver disease and non-alcoholic steatohepatitis primarily by promoting insulin resistance and fat accumulation. Ketohexokinase C (KHK-C), a pivotal enzyme in fructose metabolism, catalyzes the phosphorylation of fructose to fructose-1-phosphate, initiating a cascade of downstream metabolic processes. In contrast to glucose metabolism, KHK-C lacks negative feedback regulation, allowing the continuous phosphorylation of fructose, which leads to heightened levels of glucose, glycogen, and triglycerides in the bloodstream and liver. While targeting KHK-C offers a promising therapeutic avenue, no drugs have yet been approved for clinical use. Pfizer’s PF-06835919 has progressed to phase II trials, demonstrating a reduction in liver fat and improved insulin sensitivity, while Eli Lilly’s LY-3522348 also shows significant potential. Nonetheless, there remains a critical need for the development of novel KHK-C inhibitors that offer improved pharmacokinetics, enhanced efficacy, and superior safety profiles.

**Methods:**

In the present study, a comprehensive computational strategy was employed to screen 460,000 compounds from the National Cancer Institute library for potential KHK-C inhibitors. Initially, pharmacophore-based virtual screening was used to identify potential hits, followed by multi-level molecular docking, binding free energy estimation, pharmacokinetic analysis, and molecular dynamics (MD) simulations to further evaluate the compounds. This multi-step approach aimed to identify compounds with strong binding affinity, favorable pharmacokinetic profiles, and high potential for efficacy as KHK-C inhibitors.

**Results:**

Ten compounds exhibited docking scores ranging from −7.79 to −9.10 kcal/mol, surpassing those of the compounds currently undergoing clinical trials, PF-06835919 (−7.768 kcal/mol) and LY-3522348 (−6.54 kcal/mol). Their calculated binding free energies ranged from −57.06 to −70.69 kcal/mol, further demonstrating their superiority over PF-06835919 (−56.71 kcal/mol) and LY-3522348 (−45.15 kcal/mol). ADMET profiling refined the selection to five compounds (**1**, **2,** and **4–6**), and molecular dynamics simulations identified compound **2** as the most stable and promising candidate compared to the clinical candidate PF-06835919.

**Conclusion:**

These findings highlight compound **2** as a potent KHK-C inhibitor with predicted pharmacokinetics and toxicity profiles supporting its potential for treating fructose-driven metabolic disorders, warranting further validation.

## 1 Introduction

**FIGURE 1:**
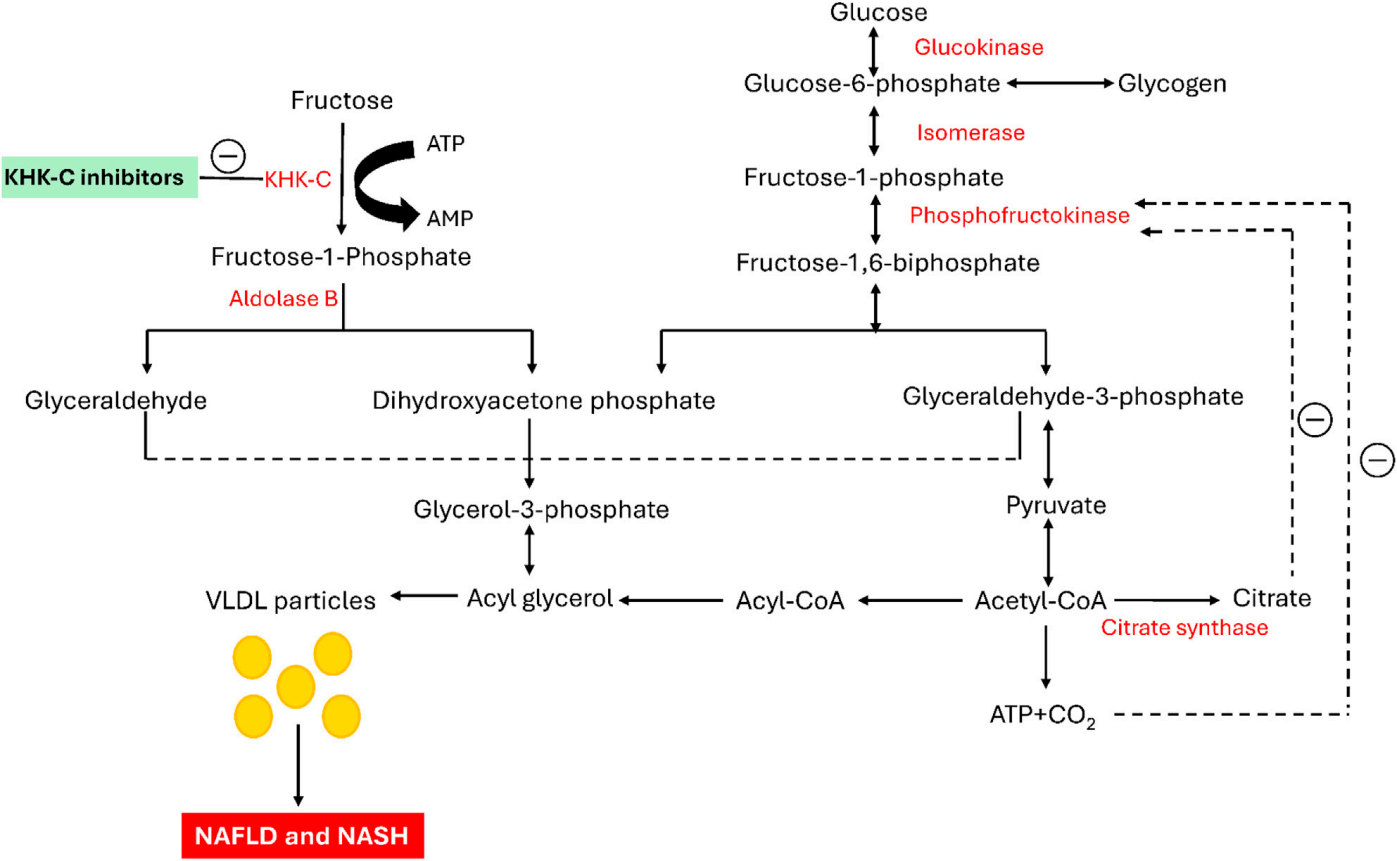
Metabolism of fructose and glucose in the liver: pathways and key enzymes involved.

The modern Westernized diet, which incorporates highly refined sugars, has been linked to various metabolic disorders, including obesity, non-insulin-dependent diabetes mellitus (NIDDM), atherogenic dyslipidemia, non-alcoholic fatty liver disease (NAFLD), and non-alcoholic steatohepatitis (NASH). (Elliott et al., 2002; Basciano et al., 2005; Jung and Choi, 2014; Kopp, 2019; Kazierad et al., 2021). Among these sugars, fructose is widely used as a sweetening ingredient in various processed food products consumed daily, such as soft drinks and desserts. (Chen et al., 2017). Diets rich in fructose induce a wide range of metabolic disturbances in both humans and animal models (Raben et al., 2002; Maryanoff et al., 2011). Fructose is primarily metabolized in the liver, where it bypasses the key regulatory checkpoint of phosphofructokinase (PFK) in glycolysis, unlike glucose (Mayes, 1993; Swarbrick et al., 2008; Stanhope et al., 2009; Eberhart et al., 2020; Zhu et al., 2023). When fructose enters the liver, it is converted by ketohexokinase into fructose-1-phosphate (F1P) utilizing adenosine triphosphate (ATP) as a cofactor (Figure 1). F1P is then split by Aldolase B into two triose sugars: glyceraldehyde and dihydroxyacetone phosphate (DHAP). These molecules are subsequently converted into glyceraldehyde-3-phosphate (G3P), which feeds into the glycolytic pathway, producing acetyl-CoA. Acetyl-CoA plays a crucial role in fatty acid synthesis, leading to the creation of triglycerides (Shepherd et al., 2021; Koene et al., 2022). These triglycerides are packaged into VLDL particles and transported into the bloodstream, increasing triglyceride levels (Zhang et al., 2011). Unlike glucose, fructose metabolism lacks regulation by high-energy signals like ATP or citrate, meaning KHK-C continues to drive the pathway without inhibition. This leads to continuous production of glycerol-3-phosphate and acetyl-CoA, contributing to excess fat accumulation in the liver. This unregulated pathway is a significant cause of non-alcoholic fatty liver disease (NAFLD), elevated blood triglycerides (hypertriglyceridemia), and increased risk of cardiovascular diseases (Jensen et al., 2018; Elsaman and Mohamed, 2025). Chronic high fructose consumption leads to insulin resistance and metabolic syndrome, both of which increase the risk of developing type 2 diabetes (Taskinen et al., 2019). Unlike glucose, which is stored as glycogen, fructose is more readily converted into fat, exacerbating metabolic issues. The overconsumption of fructose, particularly from sugary foods, underscores the importance of understanding its role in modern health challenges (Ting, 2024).

**FIGURE 2:**
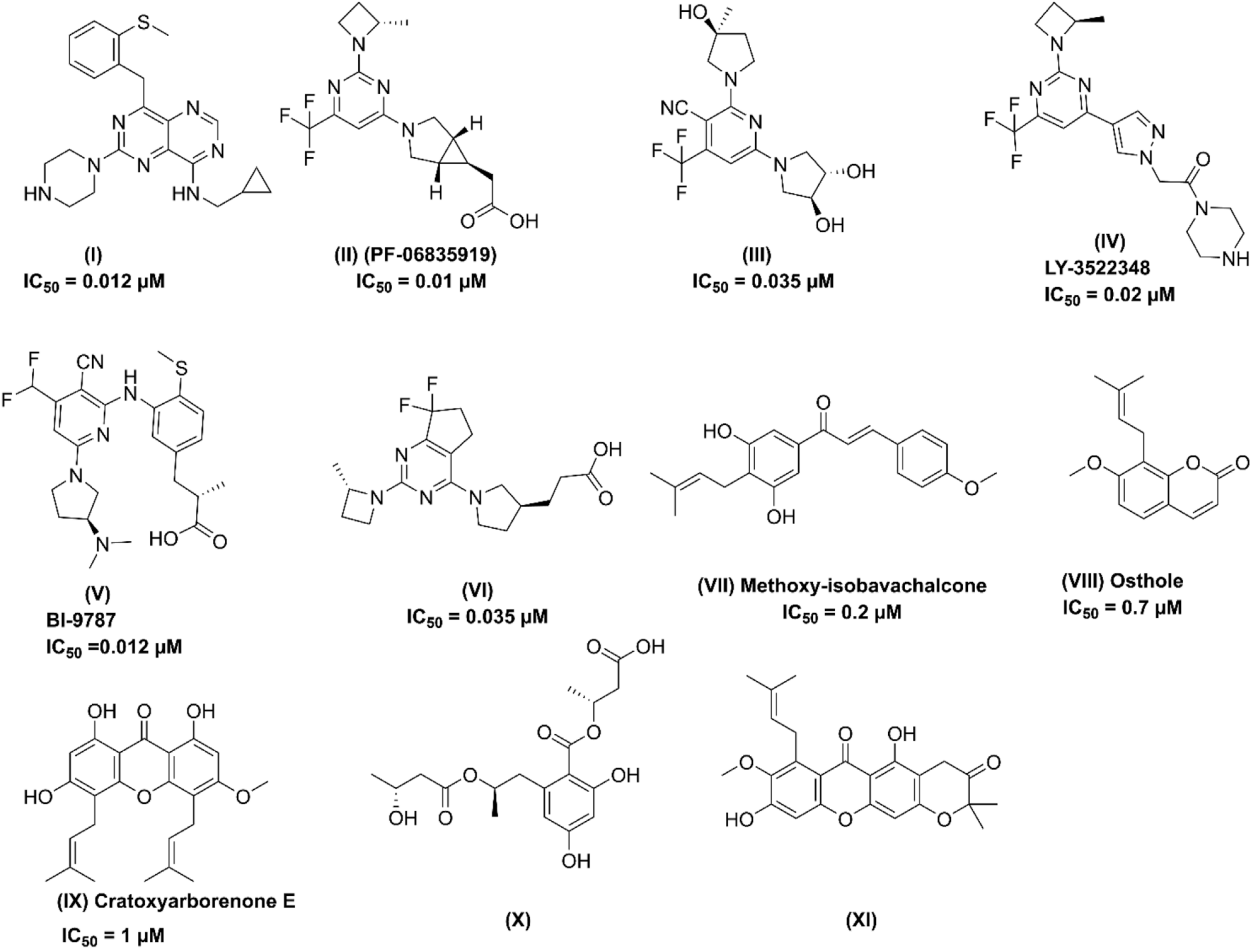
Chemical structures of some of the reported potent KHK-C isoform inhibitors.

Initially, fructose is phosphorylated by the enzyme KHK-C into fructose-1-phosphate. Subsequently, it is cleaved by Aldolase B, generating the trioses glyceraldehyde and dihydroxyacetone phosphate. The resultant glyceraldehyde is then phosphorylated to form glyceraldehyde-3-phosphate. The phosphorylated trioses enter the glycolytic pathway at this step and are ultimately converted into triglycerides and VLDL particles, promoting intrahepatic lipogenesis. Glucose metabolism is regulated by the negative feedback inhibition of phosphofructokinase by both ATP and citrate. In contrast, KHK-C lacks negative feedback control, allowing fructose to serve as an unregulated source of both glycerol-3-phosphate and acyl-CoA, leading to elevated triglyceride levels in both the blood and liver (Elliott et al., 2002). Furthermore, a study in mice by Lanaspa and colleagues highlighted the critical role of KHK-C in the pathogenesis of fructose-mediated metabolic abnormalities, including dyslipidemia and hepatic steatosis (Lanaspa et al., 2018). Similarly, Ishimoto et al. demonstrated that KHK-null mice were protected from the negative metabolic effects of a high-fructose diet, highlighting the critical role of KHK in the development of fructose-induced metabolic syndrome (Ishimoto et al., 2012). In light of these nonclinical studies, KHK inhibition is emerging as a potential therapeutic strategy for treating fructose-related metabolic disorders (Huard et al., 2017; Liu et al., 2018; Gutierrez et al., 2021; Kazierad et al., 2021; Shepherd et al., 2021). KHK plays a crucial role in the initial stage of fructose metabolism and is predominantly found in the liver, kidney, and brain, although it is present in various other tissues. A deficiency in hepatic KHK leads to essential fructosuria, a benign disorder, indicating that KHK may be a potential target for therapeutic intervention. Inhibiting hepatic KHK could prevent the metabolism of fructose (which constitutes 50% of dietary sucrose) and have a positive impact on various metabolic processes (Herman and Birnbaum, 2021). KHK exists in two isoforms, KHK-A and KHK-C, with KHK-C serving as the predominant enzyme responsible for fructose metabolism in the liver. This dominance is attributed to its significantly lower *K*_*M*_ and higher V_max_ values compared to KHK-A, indicating greater catalytic efficiency (Ishimoto et al., 2012; Gutierrez et al., 2021; Ferreira et al., 2024). KHK-C is predominantly expressed in key organs involved in fructose metabolism, such as the liver, kidney, and intestine, whereas KHK-A is found at lower levels across various tissues (Diggle et al., 2009). Both enzymes exhibit catalytic activity, and there is a high degree of homology at the active site. While several pharmacological treatments have been approved to manage excess glucose in the body, no therapies specifically targeting fructose have been identified (Durham et al., 2023). Previous efforts have focused on developing potent small-molecule inhibitors of the KHK-C isoform, derived from chemically distinct heterocycles, through synthetic approaches, *in silico* studies, and natural product exploration, as reported in the literature. Several of these molecules have demonstrated potent inhibitory activity against the KHK-C isoform (Maryanoff et al., 2011; Huard et al., 2017; Futatsugi et al., 2020; Durham et al., 2023; Zhu et al., 2023). Among them, compound **I** (Figure 2) has been identified as the most potent KHK inhibitor to date. However, pharmacokinetic studies in rats revealed low exposure levels, likely due to rapid metabolic clearance (Zhu et al., 2023). Compound **II** (PF-06835919) (Figure 2), developed by Pfizer through the optimization of lead compound **III**, has become a top lead in Phase II clinical trials for the treatment of NAFLD (Futatsugi et al., 2020; Zhu et al., 2023). Research indicates that this compound induces a pharmacodynamic response in human patients, leading to a reduction in liver fat and enhanced insulin sensitivity (Gutierrez et al., 2021). Another notable clinical candidate for KHK inhibition is compound **IV** (LY-3522348) (Figure 2), introduced by Eli Lilly and Company in 2020, which demonstrated strong efficacy (Durham, 2020; Durham et al., 2023). Further, Heine et al. recently identified compound **V** (BI-9787) (Figure 2), a potent zwitterionic KHK inhibitor known for its high permeability and favorable oral pharmacokinetic characteristics in rats (Heine et al., 2024). Similarly, authors from TuoJie Biotech recently reported another structurally related KHK inhibitor (compound **VI**), which shows potential for clinical development (Zhu et al., 2023). Furthermore, exploration of potential KHK-C inhibitors has extended beyond synthetic compounds to include natural products, which have shown promising activity in modulating this enzyme. Among the purified phytochemicals (Figure 2), methoxy-isobavachalcone **(VII)** from *Psoralea corylifolia* showed the strongest inhibition of ketohexokinase isoform C, followed by osthole **(VIII)** from *Angelica archangelica*, and cratoxyarborenone E **(IX)** from *Cratoxylum prunifolium*, (Le et al., 2016). Additionally, computational studies utilizing structure-based approaches were performed to identify potent inhibitors. For example, Alturki applied an *in silico* method to discover potential KHK-C inhibitors from natural marine organisms, with compound **X** identified as the top binders (Alturki, 2024). Furthermore, prenylated xanthone (**XI**), as demonstrated by Elsaman et al., exhibited a high binding affinity towards KHK-C (Figure 2) (Elsaman and Mohamed, 2025). Further, Tiwari et al. recently identified the natural products saroglitazar and ferulic acid as potential inhibit using *in silico* methods (Tiwari et al., 2025). Despite these efforts, no drugs directly targeting the KHK-C isoform have been clinically approved, and only PF-06835919 and LY-3522348 have reached phase II clinical trials (Durham et al., 2023; Zhu et al., 2023).

**FIGURE 3:**
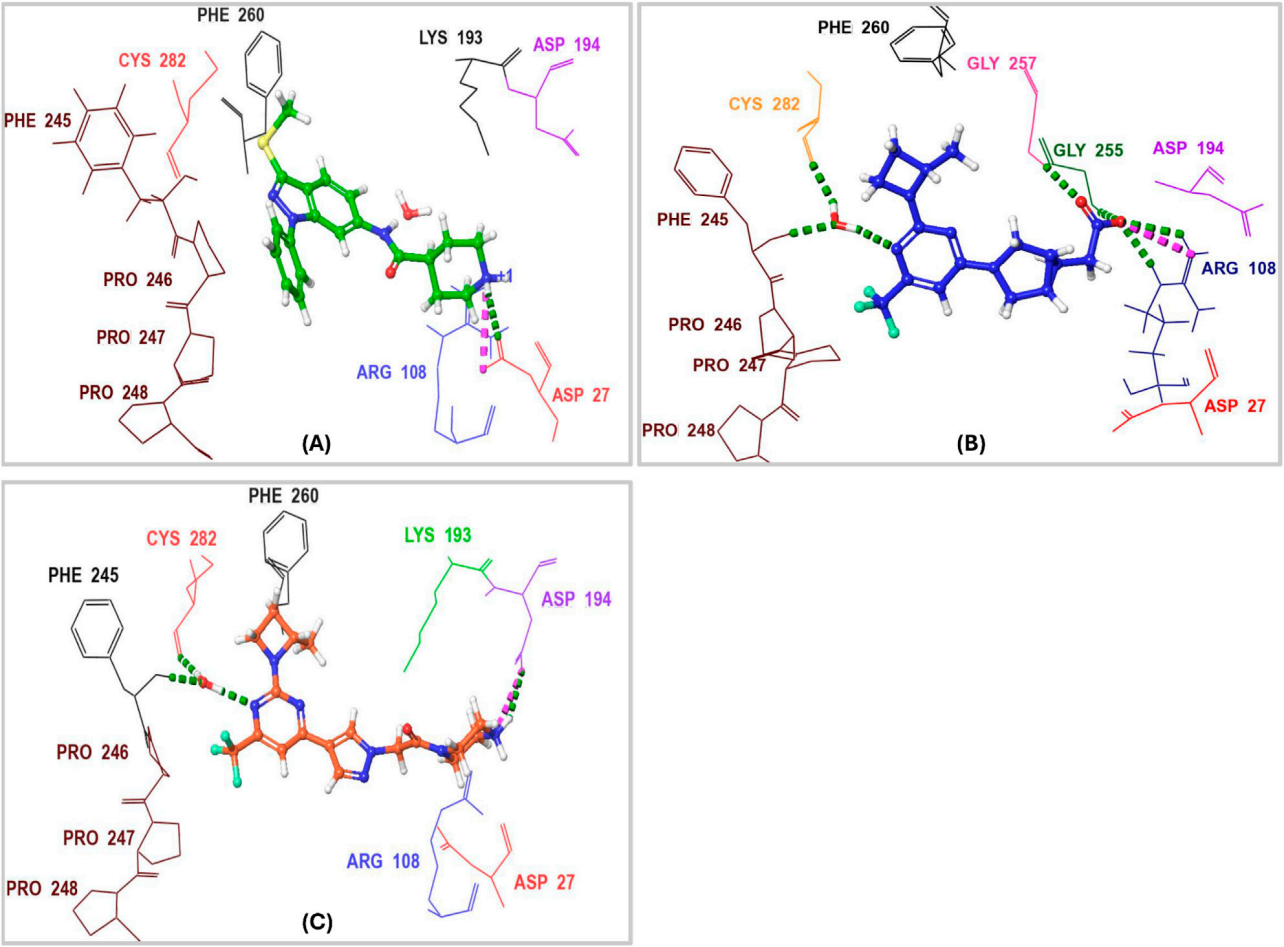
3D Interaction Diagram of Human KHK-C in Complex with PF-06835919, LY-3522348, and an Indazole Derivative. **(A)** Binding mode of the indazole derivative within the ATP-binding pocket of KHK-C. **(B)** Binding mode of PF-06835919 within the ATP-binding pocket of KHK-C. **(C)** Binding mode of LY-3522348 within the ATP-binding pocket of KHK-C. Amino acid residues are labeled using three-letter codes. Hydrogen bonds are represented in green, while salt bridges are depicted in pink.

Characterizing the interactions between ketohexokinase-C (KHK-C) and its inhibitors is crucial for advancing drug design targeting fructose metabolism disorders. To investigate these interactions, the binding modes of human KHK-C in complex with three previously reported inhibitors PF-06835919 (PDB ID: **6W0Z**), LY-3522348 (PDB ID: **8UG3**), and an indazole derivative (PDB ID: **3NC9**) were examined (Figure 3) (Gibbs et al., 2010; Futatsugi et al., 2020; Durham et al., 2023). This analysis helps identify key binding features that can guide the development of more effective KHK-C inhibitors. All three inhibitors PF-06835919, LY-3522348, and the indazole derivative occupy the ATP-binding pocket at the dimeric junction of chains A and B in the KHK-C enzyme. The pyrazole ring of the indazole derivative (Figure 3A) and the (S)-2-methylazetidine groups in PF-06835919 (Figure 3B) and LY-3522348 (Figure 3C) occupy the ATP-ribose pocket, with their methyl groups fitting into a small but essential sub-pocket defined by Phe260, which typically accommodates the methylene of the ATP ribose sidechain. Additionally, the N1 of the pyrimidine core in PF-06835919 and LY-3522348 forms a hydrogen bond with a conserved water molecule that interacts with the backbone NH of Phe245 and the backbone CO of Cys282. However, these interactions are absent in the indazole derivative, which instead forms a hydrogen bond and a salt bridge between the positively charged nitrogen of its piperidine ring and Asp27 from chain B. Similarly, LY-3522348 establishes hydrogen bonds and a salt bridge between its positively charged N4 of the piperazine ring and Asp194. In contrast, PF-06835919 engages in multiple polar interactions through its ionized carboxylate group, forming hydrogen bonds with Gly255 and Gly257, as well as a salt bridge with the positively charged center of Arg108, facilitating polar interactions at the solvent-exposed area of the ATP binding pocket. Further, the trifluoromethyl groups at C6 in PF-06835919 and LY-3522348, along with the phenyl ring attached to N1 of the pyrazole ring in the indazole derivative, are positioned within a hydrophobic pocket formed by the proline loop Pro246–Pro248. A similar interaction pattern has been reported for BI-9787, a potent zwitterionic ketohexokinase inhibitor. However, its crystal structure (PDB ID: **9FHD**) has not yet been deposited in the Protein Data Bank (PDB) (Heine et al., 2024).

Considering the urgent market need for effective treatments for both NAFLD and NASH ((Elliott et al., 2002; Chen et al., 2017; Romero et al., 2020; Skenderian et al., 2020; Zhu et al., 2023), the discovery of potent inhibitors targeting the KHK-C isoform remains imperative. Despite the availability of the 3D experimental structure of KHK-C, a literature review revealed a very limited number of studies utilizing combined computational methods to identify potential KHK-C isoform inhibitors (Maryanoff et al., 2011; Huard et al., 2017; Zhu et al., 2023). Furthermore, to the best of our knowledge, no attempt has been made to screen large virtual libraries for potential hits targeting the KHK-C isoform. Therefore, this study aims to comprehensively apply multiple computational approaches, including molecular docking, pharmacophore modeling, molecular dynamics (MD) simulations, and MM-GBSA calculations, to screen the NCI library of 460,000 small molecules. The primary objective was to identify novel, high-affinity KHK-C isoform inhibitors with favorable pharmacokinetic and drug-likeness properties, which could serve as potential leads for further experimental validation and drug development efforts.

## 2 Materials and methods

**FIGURE 4:**
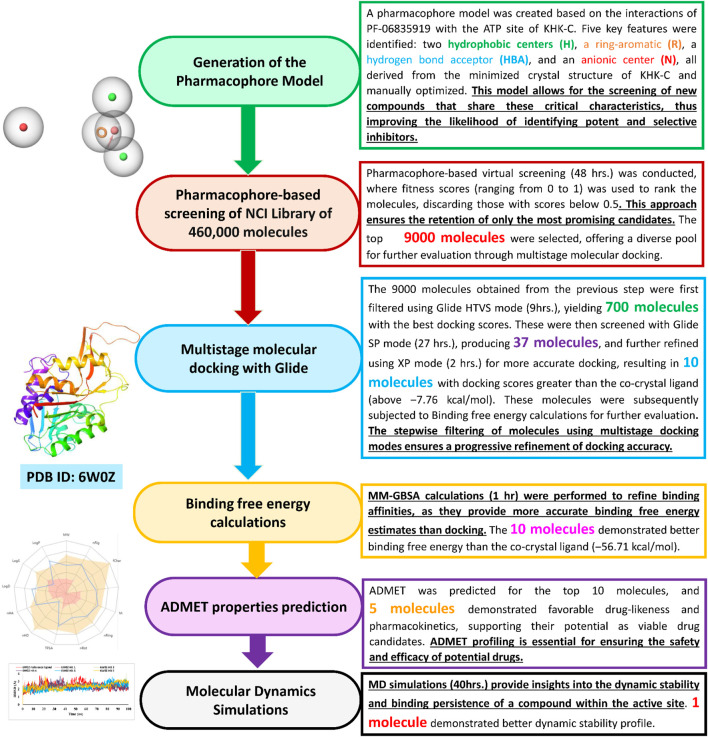
Virtual screening workflow for identification of KHK-C isoform inhibitors from a small molecules database.

The overall methodology for our research work has been provided by the flow chart for identifying KHK-C isoform inhibitors from the NCI library is illustrated in Figure 4, with each step accompanied by its underlying rationale. All of our research was conducted using the commercial Schrodinger Suite, developed by Schrödinger, LLC (www.schrodinger.com). This suite includes a variety of powerful programs such as Glide, Maestro, Phase, Desmond, Prime, and QikProp, which were utilized for molecular modeling, docking, pharmacophore generation, molecular dynamics simulations, protein structure preparation, and ADMET predictions. The suite consists of several advanced tools, each with its specific functions. Glide is a molecular docking software that predicts how small molecules bind to protein targets, providing accurate docking results and scoring. Maestro acts as the interface for Schrödinger’s computational chemistry tools, enabling molecular modeling, visualization, and simulation tasks. Phase specializes in pharmacophore modeling, identifying essential molecular features that interact with biological targets, which is crucial for drug discovery. Desmond is a molecular dynamics simulation tool that analyzes the movement of atoms and molecules over time, aiding in the study of protein-ligand interactions and conformational changes. Prime is used to refine and predict protein structures, improving the precision of 3D models of proteins and peptides. Lastly, QikProp is designed to predict ADMET (Absorption, Distribution, Metabolism, Excretion, and Toxicity) properties, helping to evaluate the drug-likeness and pharmacokinetics of compounds.

### 2.1 The target protein preparation

The` crystal structure of the human KHK-C protein, complexed with the small molecule PF-06835919, was obtained in PDB format from the freely accessible RCSB Protein Data Bank (https://www.rcsb.org/) (accessed on 8 July 2023), PDB ID: **6W0Z**, with a resolution of 2.30 Å (Futatsugi et al., 2020). The structure consists of two chains, A and B, from which chain B was selected for further analysis. Subsequently, the protein structure was processed using the Protein Preparation Wizard (Protein Preparation Workflow - Schrödinger) module of Schrödinger Maestro software (2023-1, http://www.schrodinger.com/) (Madhavi Sastry et al., 2013). The Protein Preparation Wizard assists in detecting and rectifying structural issues by adding hydrogen atoms, completing missing side chains, and assigning proper bond orders. The ionization and tautomeric states of amino acid residues were adjusted by incorporating hydrogen atoms. Epik was used to determine the protonation states of heteroatoms, ensuring that hydrogen atoms were assigned as they would occur at a physiological pH of 7.0 ± 2.0. Water molecules located beyond 3.0 Å from the ligand were removed. The downloaded protein underwent multiple steps, including import and refinement, structural review and modifications, and energy minimization. Missing residues and side chains were reconstructed using the Prime tool, ensuring that key active sites remain unaltered. Energy minimization was performed with an RMSD cutoff of 0.30 Å using the OPLS4 (Optimized Potential for Liquid Simulations) force field to achieve a stable, low-energy conformation of the protein (Lu et al., 2021). The optimized structures were saved in Maestro (.mae) format for further predictive analysis.

### 2.2 Structure-based pharmacophore model generation and pharmacophore-based virtual screening

**FIGURE 5:**
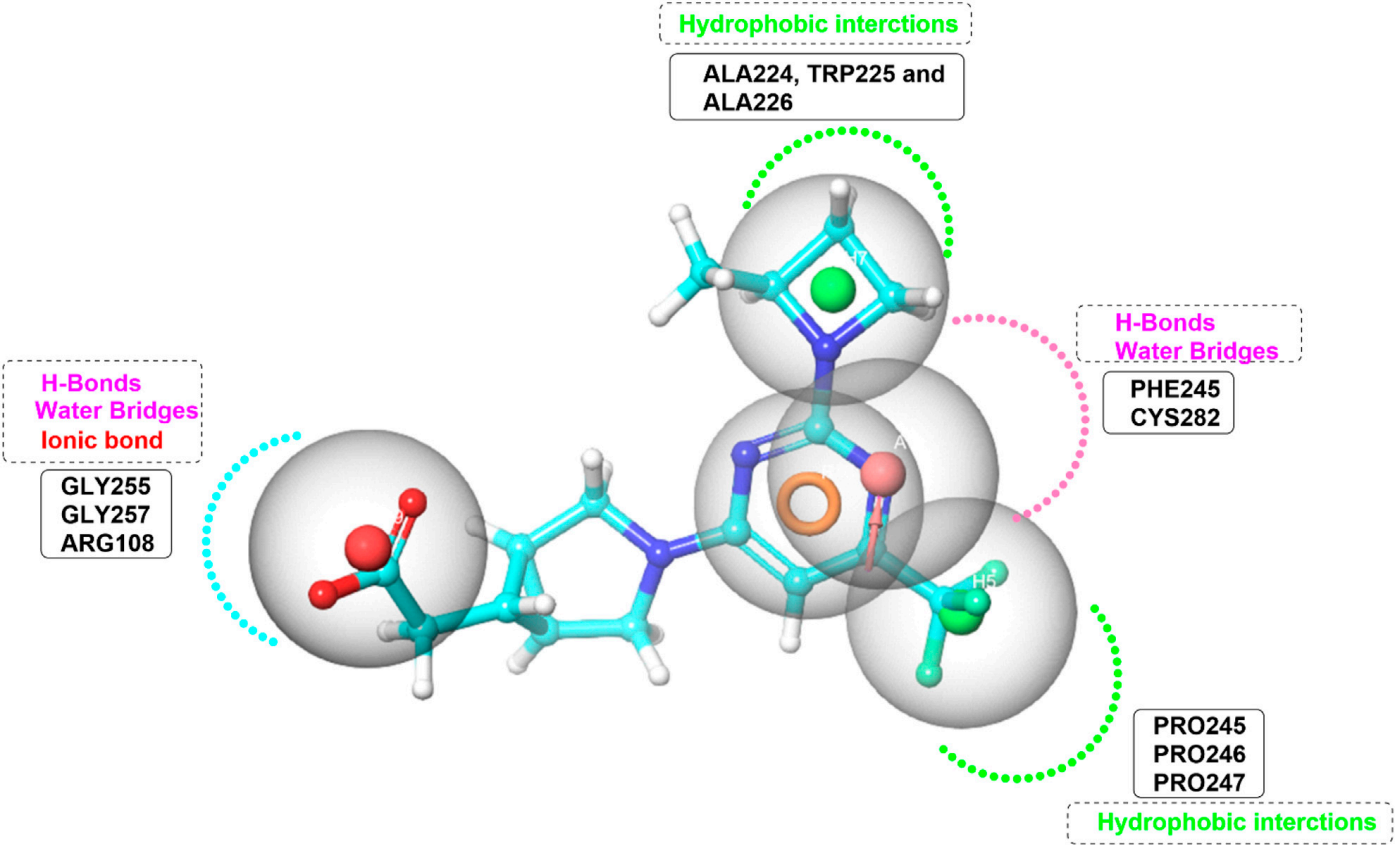
Pharmacophore Model Depicting Interaction Forces and Residues in the KHK-C Binding Site. Hydrophobic interactions (green color), Hydrogen bond acceptors (HBA) (pink color), and ionic bond (red color).

Pharmacophore model was generated using the Phase module (Schrödinger Press, 2024) from Maestro (Dixon et al., 2006a; Dixon et al., 2006b). The pharmacophore model was systematically designed based on the critical interactions of the potent ATP-competitive inhibitor PF-06835919 within the ATP-binding site of KHK-C (Figure 5). To ensure the model accurately represents the essential molecular interactions required for effective inhibition, five key pharmacophoric features were identified. Two hydrophobic (H) features were incorporated: one corresponding to the azetidine ring, which interacts with the hydrophobic pocket formed by Ala224, Trp225, and Ala226, and the other corresponding to the trifluoromethyl group, which engages with the proline loop composed of Pro246, Pro247, and Pro248. A ring-aromatic (R) feature was assigned to the pyrimidine core to mimic the structural positioning of the adenine moiety in ATP within the active site. A hydrogen bond acceptor (HBA) feature was positioned at the nitrogen of the pyrimidine ring, replicating the hydrogen bonding interactions with Cys282 and Pro245 through water bridges. Lastly, an anionic center (N) was included to represent the carboxylate functional group, forming an essential salt bridge with Arg108, a conserved residue involved in the catalytic mechanism of KHK-C. In developing the pharmacophore model, the importance of assigning feature requirements and permissiveness was carefully considered to ensure the accurate prediction of ligand-target interactions. Features such as the ring and hydrophobic centers were assigned as required based on their consistent presence in all active compounds, while the negative center and hydrogen bond acceptor were assigned as permitted, given their frequent occurrence in a significant proportion of the active ligands. These features were generated from the minimized crystal structure of KHK-C and then manually optimized. Exclusion volume spheres were executed and all the rest parameters in the program were kept as default. Pharmacophore-based virtual screening was carried out using Phase screening protocol impeded in Schrödinger software (http://www.schrodinger.com/). Enrichment analysis was performed on a dataset of 45 ligands, including 30 active compounds sourced from literature (Maryanoff et al., 2011; Huard et al., 2017; Durham et al., 2023; Zhu et al., 2023; Heine et al., 2024). The analysis utilized the Balanced Successive Early Recognition Operating Characteristic (BEDROC), Receiver Operating Characteristic (ROC), Area Under the Curve (AUC) and Enrichment Factor (EF) metrics to assess early active recovery and hit rates were calculated for the top N% of results. The generated model represented the spatial arrangement of key features required for KHK-C binding The Open National Cancer Institute (NCI) database (https://cactus.nci.nih.gov/download/roadmap/(accessed on 31 August 2023) was chosen for screening due to its extensive collection of structurally diverse and bioactive compounds, increasing the chances of identifying potential inhibitors. Its publicly available and well-curated data ensures transparency and reproducibility. Many compounds have known biological activity, making them valuable for drug repurposing. The database facilitates structure-activity relationship (SAR) studies, aiding in lead optimization. Furthermore, Its relevance to drug discovery further supports the identification of effective KHK-C inhibitors (Voigt et al., 2001). For each molecule in the NCI library, 50 conformers were generated and the PhaseScreenScore (fitness score) values (range from 0 to 1) were used to rank the molecules. This score assesses the alignment of key vector features such as acceptors, donors, and aromatic rings with the pharmacophore model, as well as the overall structural overlap with the reference ligand. A maximum of 0.5 Å RMSD (Root Mean Square Deviation) from sphere centers was used as an input parameter for the prepared library. Molecules displayed values less than 0.5 were rejected and the top scored 9,000 molecules were submitted to multistage molecular docking with Glide. All adjustable parameters were maintained at their default settings. Based on the mapping of pharmacophore features.

### 2.3 Multistage molecular docking and MM-GBSA calculations

The virtual screening was performed using Glide (Schrödinger Press, 2024) in the Schrödinger suite (Halgren et al., 2004; Friesner et al., 2004; Friesner et al., 2006). The docking protocol was validated by redocking PF-06835919 into the active site of KHK-C (PDB ID: **6W0Z**). The docking prediction was considered successful when the Root Mean Square Deviation deviation (RMSD) of the best-scored conformation was less than 2.0 Å. Initially, the top-ranked 9,000 molecules obtained from the previous step were prepared using Ligprep module of Schrödinger suite (LigPrep, 2023). The original chirality of these molecules was retained and the potential ionization forms were developed at pH 7.00 ± 2 units using Epik. Subsequently these molecules were minimized using OPLS4 force field (Lu et al., 2021) and a low energy conformer were generated for each ligand. The grid box generated around the reference ligand was used to dock the prepared molecules into the KHK-C binding site. This grid box was specifically designed to encompass the entire binding site, ensuring accurate molecular placement. We utilized the receptor-based grid box generation tool in Schrödinger, with the default settings applied. Next, the prepared ligands were submitted to Virtual Screening Workflow (VSW) utility module within the Schrödinger molecular modeling suite employing Glide software (Halgren et al.; Friesner et al., 2004; Friesner et al., 2006). The VSW protocol mainly involves three sequential docking methods, where the outcome of each method is used as the input for the subsequent one. In each docking step, we selected the top 10% of the molecules. The 9,000 molecules were firstly filtered using Glide high-throughput virtual screening (HTVS) mode. This was followed by screening with standard precision (SP) mode. Lastly, Glide extra precision (XP) docking algorithm was utilized for more accurate docking calculations results. During the docking process, the potential for the nonpolar regions of the target was softened by adjusting the scaling factor of the van der Waals radii to 0.80, with a cut-off value of 0.15, alongside the use of other default parameters. One best pose was generated for each docked molecule and the Glide XP docking score was used to select the best molecules. Further, enrichment analysis was performed to validate the docking protocol by distinguishing known KHK-C inactives, sourced from the literature (Maryanoff et al., 2011; Le et al., 2016; Huard et al., 2017; Zhu et al., 2023) with IC_50_ values greater than 1 μM, from active compounds. With IC_50_ values greater than 1 μM, from active compounds. The performance was assessed using a Receiver Operating Characteristic (ROC) curve, which evaluated the ability of the docking protocol to correctly rank actives over inactives. To assess the consistency and robustness of the docking results, cross-validation was further performed using AutoDock 4.2, a freely available software program (http://autodock.scripps.edu/). The docking procedure involved preparing the ligand and protein structures, followed by grid generation and optimization of the docking parameters. The binding affinities of the identified hits were calculated based on the docking scores. 3D and 2D molecular interactions modes were generated using Maestro interface of Schrödinger suite. The obtained best 10 molecules based on their docking score were selected for further estimation of their binding free energies. Prime module (Prime | Schrödinger) (Jacobson et al., 2002; Jacobson et al., 2004) interfaced with Schrödinger suite was used to calculate the molecular mechanics-generalized Born surface area (MM-GBSA) free energy for each molecule. The Post docking generated Pose Viewer File (PVF) for each of the top-ranked 10 molecules was employed as input file for energy computation. The VSGB 2.0 solvation model and OPLS4 force field (Lu et al., 2021) were utilized to calculate free energy parameters, based on a previously reported protocol (Elsaman et al., 2024). Ligands were ranked using the MMGBSA ∆G binding energy score. Compounds that exhibited higher XP docking scores and binding energies compared to the co-crystal ligand were subjected to *in silico* pharmacokinetics analysis. The 2D and 3D binding interactions, essential for visualizing molecular structures, were generated using the advanced tools and capabilities offered by the Schrödinger Maestro interface.

### 2.4 ADMET profiling

The drug-likeness evaluation is an essential and fundamental step in selecting potential compounds from a gathered chemical dataset. The ADMET profiles and drug-likeness descriptors of the top 10 hits, identified through their high MMGBSA ∆G binding energy scores, were assessed using the computational tool QikProp (Schrödinger Release 2023-1) (QikProp Schrödinger) (Laoui and Polyakov, 2011). Each calculated descriptor was evaluated to ensure compliance with the permissible ranges. The ligand structures were prepared with LigPrep to optimize their 3D structures and assign protonation states at a physiological pH of 7.4. The QikProp tool, using its default settings, predicted various parameters such as LogP, solubility (LogS), CNS permeability, human oral absorption, and toxicological risks. All calculations were performed in standalone mode within the Schrödinger software environment. QikProp provides recommended ranges for molecular properties, based on an analysis of 95% of known drugs. The results, including key descriptors and ADME predictions, were exported into an MS Excel file for further analysis.

### 2.5 Molecular dynamics (MD) simulations

After the selection of the top five docked complexes (molecules 1, 2, 4–6) based on Glide XP scores, binding energy evaluations, and favorable ADMET and physicochemical properties, molecular dynamics (MD) simulations were conducted to assess the stability of the interactions between the selected compounds and the KHK-C binding site. These simulations were compared to the performance of the co-crystal ligand PF-06835919. To provide a more comprehensive analysis, the study also included simulations with the apoprotein, allowing for a better understanding of the behavior and interactions of both the unbound protein and protein-ligand complexes in physiological conditions. MD simulations, which typically predict ligand binding rankings by solving Newton’s classical equations of motion (Hildebrand et al., 2019). The simulations were carried out using the Desmond package (Desmond Schrödinger Life Science) from Schrödinger LLC (Bowers et al., 2006). A 100 ns classical all-atom MD simulation was conducted on a Linux (Ubuntu) desktop server equipped with GPU-enabled Schrodinger Desmond software to investigate the stability of the top-ranked complexes docked with KHK-C. The complexes were placed in an orthorhombic box with 10 Å × 10 Å × 10 Å dimensions and solvated with the Simple Point Charge (SPC) water model. A Na + counterion was introduced to neutralize the system, and additional 0.15 M NaCl was added to mimic the physiological ionic strength. The default relaxation protocol was applied, including energy minimization and pre-equilibration steps to refine and optimize the energy of the complexes. This protocol incorporated restrained minimization, followed by gradual relaxation of the solvated system, employing OPLS4 force field parameters (Lu et al., 2021) to eliminate steric clashes, weak interactions, and distorted geometries. The system was maintained under physiological conditions during the simulation at 310 K and 1 bar using the NPT ensemble. Structures were optimized for enhanced flexibility before the simulation, with trajectories saved every 100 ps for subsequent analysis. Upon completion, parameters such as Root Mean Square Deviation (RMSD), Root Mean Square Fluctuation (RMSF), and protein-ligand interactions were extracted from the MD trajectories and analyzed using Desmond’s event analysis module. The stability of the interactions, including hydrogen bonding, hydrophobic contacts, and π-π stacking, was monitored throughout the simulation. For visualization of the simulation events and interactions, the Simulation Interaction Diagram (SID) tool in Maestro was used.

## 3 Results

### 3.1 Structure-based pharmacophore model generation and pharmacophore-based virtual screening

Pharmacophore modeling is most commonly used in virtual screening to identify compounds that demonstrate the desired pharmacological action (Voet et al., 2014). In the present study, the X-ray crystal structure of KHK-C (PDB: **6W0Z**) in complex with compound PF-06835919 (IC_50_ value 0.01 µM) was retrieved from the Protein Data Bank. The protein crystal structure has a resolution of 2.30 Å.

In this study, we performed hypothesis validation to evaluate the generated hypothesis for identifying potential KHK-C inhibitors. Key validation metrics demonstrated strong classification performance between actives and decoys (Supplementary Figure S1). The RIE (Ranking Information Entropy) was 1.50, indicating that actives were ranked significantly higher than decoys. The AUC value was 0.62, reflecting effective accumulation of actives over time. BEDROC values for different alpha parameters (160.9, 20.0, and 8.0) were 1.000, 1.000, and 0.977, respectively, supporting the model’s capability to prioritize actives in the ranking list. In the screening results (Supplementary Figure S1), 56.7% of the actives were ranked within the top 20% of the decoys, showing significant enrichment. The Enrichment Factor (EF) for different sample sizes, including 1%, 2%, and 5%, remained consistently high, with EF' values confirming favorable enrichment. The hit rate among the top 50 ligands was 52%, and the average number of outranking decoys was 1. The EF value for recovering 50% of actives was 7.5, further confirming the model’s predictive strength. These results indicate that the hypothesis is effective in identifying potential KHK-C inhibitors.

The pharmacophore model identified key features essential for ligand binding. The ring feature and two hydrophobic features were present in all retrieved active compounds, leading to their classification as required. The negative center was observed in 14 out of 26 retrieved actives (53.8%) and was assigned as permitted. The hydrogen bond acceptor feature appeared in 13 out of 26 actives (50%) and was also assigned as permitted. The finalized pharmacophore model was used to screen the prepared NCI library, yielding 9,000 selected molecules for structure-based virtual screening. These compounds were ranked based on phase fitness scores to evaluate their potential as active inhibitors. The selected candidates were further analyzed for their ability to interact with the target.

### 3.2 Multistage molecular docking and MM-GBSA calculations

**FIGURE 6:**
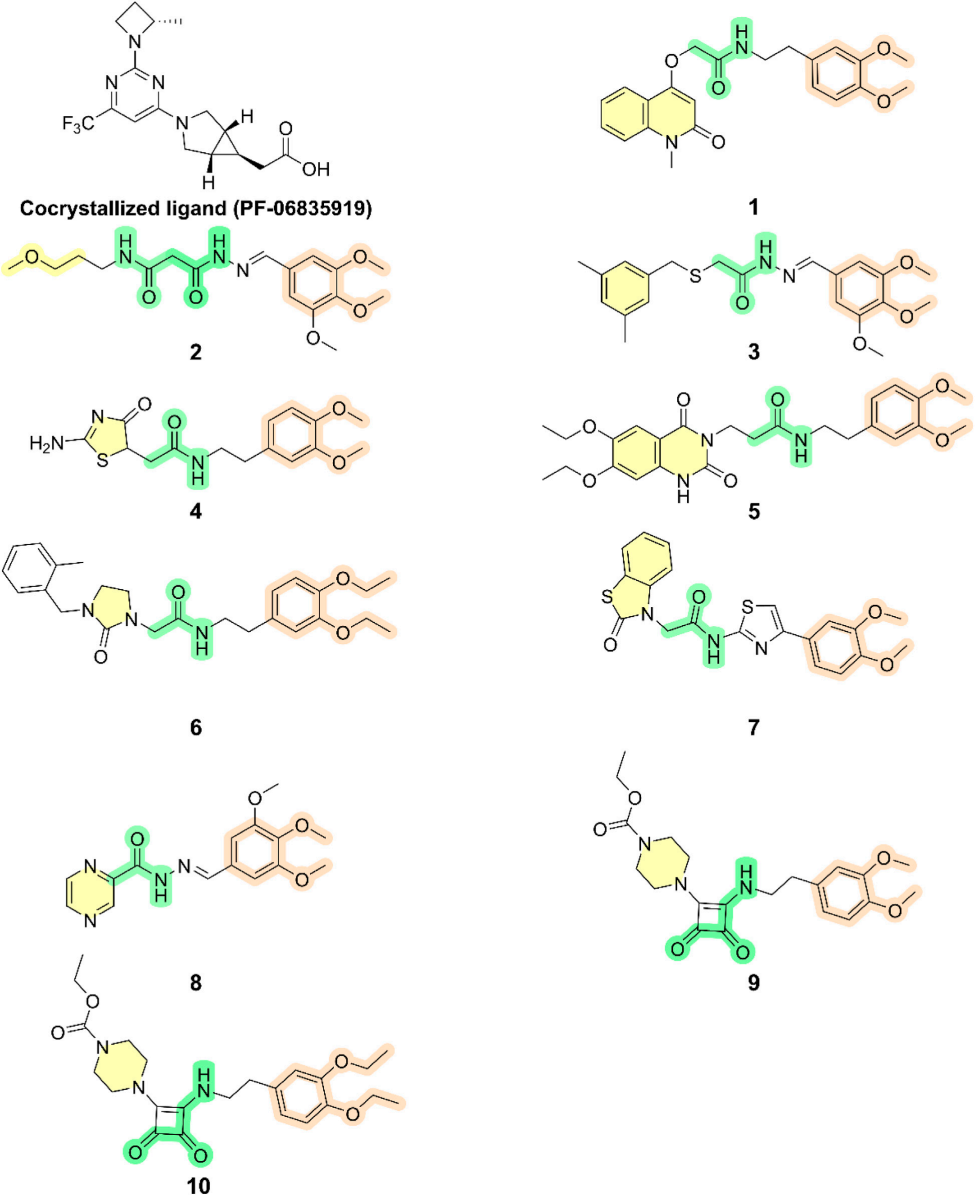
Chemical structures of the top 10 hits and the co-crystallized ligand (PF-06835919).

Pharmacophore-based virtual screening retrieved over 9,000 compounds. High-throughput virtual screening (HTVS) reduced this to 700 compounds, followed by standard precision (SP) docking, which identified 37 compounds with better docking scores than the reference ligand. Glide extra precision (XP) docking further refined the selection to 10 hits with docking scores between −7.79 and −9.10 kcal/mol, all outperforming the co-crystallized ligand (−7.76 kcal/mol). Hit 6 had the highest docking score (−9.10 kcal/mol). The chemical structures of the top-ranked hits are shown in Figure 6.

**FIGURE 7:**
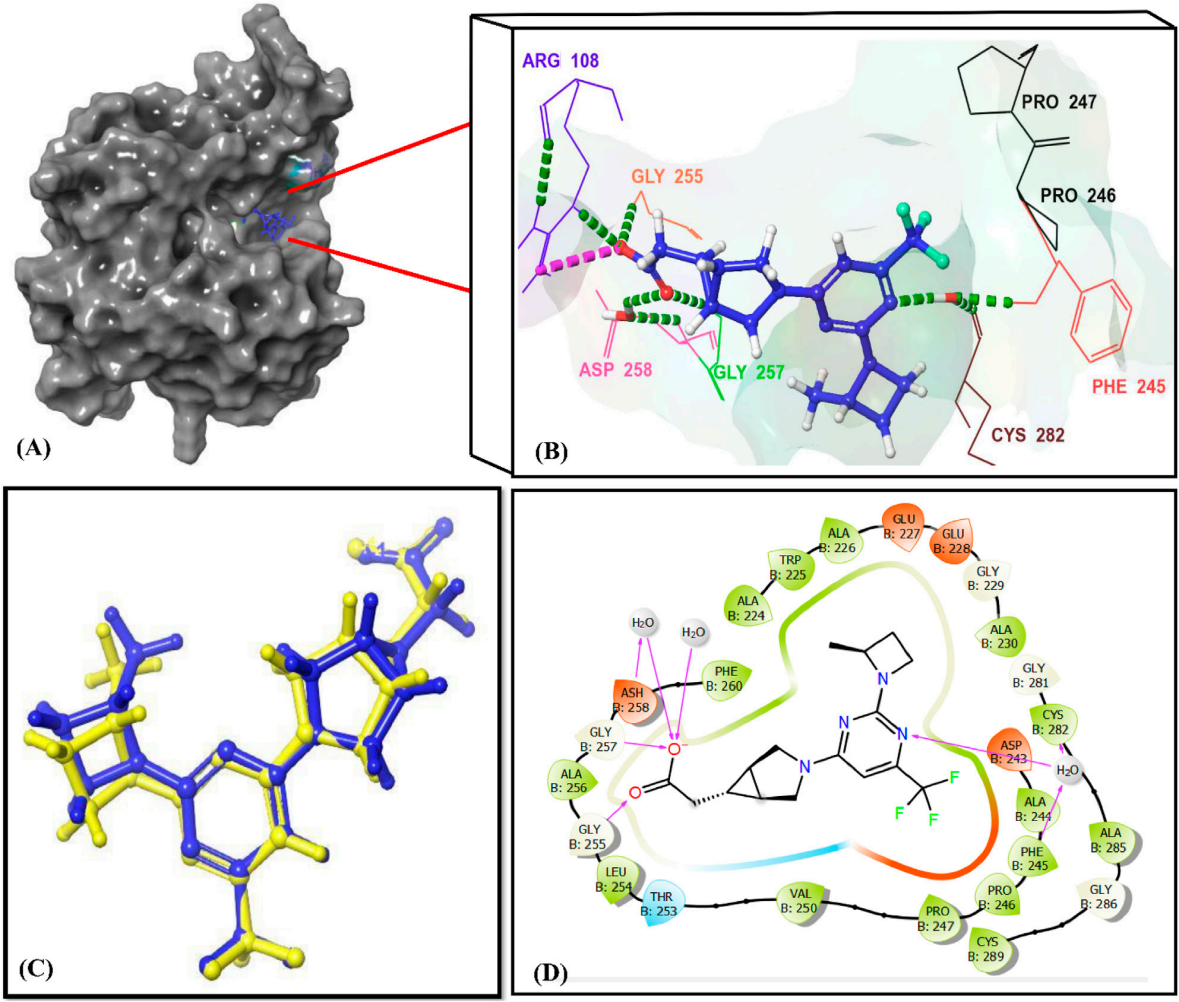
Interactions of the reference ligand PF-06835919 with the KHK-C binding site (PDB ID: 6W0Z). Panel **(A)** shows the crystal structure of the target enzyme bound to PF-06835919. Panels **(B, D)** depict the 3D and 2D enzyme-ligand interactions, respectively. In Panels (B, D), the binding site residues (Phe245, Pro246, Pro247, Pro248, Gly255, Gly257, ASH258, and Cys282) are indicated using their three-letter amino acid codes. H-Bonds are represented by green dotted lines in Panel (B) and by magenta lines in Panel **(D)**. Panel **(C)** illustrates the superimposition of the docked reference ligand PF-06835919 (in blue) with the original ligand (in yellow), showing an RMSD value of 0.245 Å.

The docking protocol was validated by re-docking the co-crystallized ligand into the same binding pocket. The Root Mean Square Deviation (RMSD) between the docked conformation and the native ligand was low (0.245 Å) (Figure 7), confirming the reliability of the docking protocol used in this study.

Enrichment analysis was performed using the top 10 hits as actives and 18 inactives (IC_50_ > 1 µM) from the literature. The chemical structures of the inactives are provided in Supplementary Figure S2, along with the ROC curve and % screen figure for visual reference. The screening workflow demonstrated high accuracy, achieving a perfect ROC score of 1.0 (Supplementary Figure S3), confirming flawless discrimination between actives and inactives. BEDROC values of 1.0 highlighted optimal early recognition of actives, while a relative initial enrichment (RIE) of 4.54 validated strong separation between active and inactive compounds. The area under the accumulation curve (0.89) demonstrated high ranking efficiency. The enrichment factor (EF = 4.6) remained stable across all sample sizes, ensuring reliability. Notably, 100% of actives were ranked within the top 20% (Supplementary Figure S3), with no actives outranked by inactives. These results confirm the robustness of the virtual screening workflow in accurately identifying potential KHK-C inhibitors.

**FIGURE 8:**
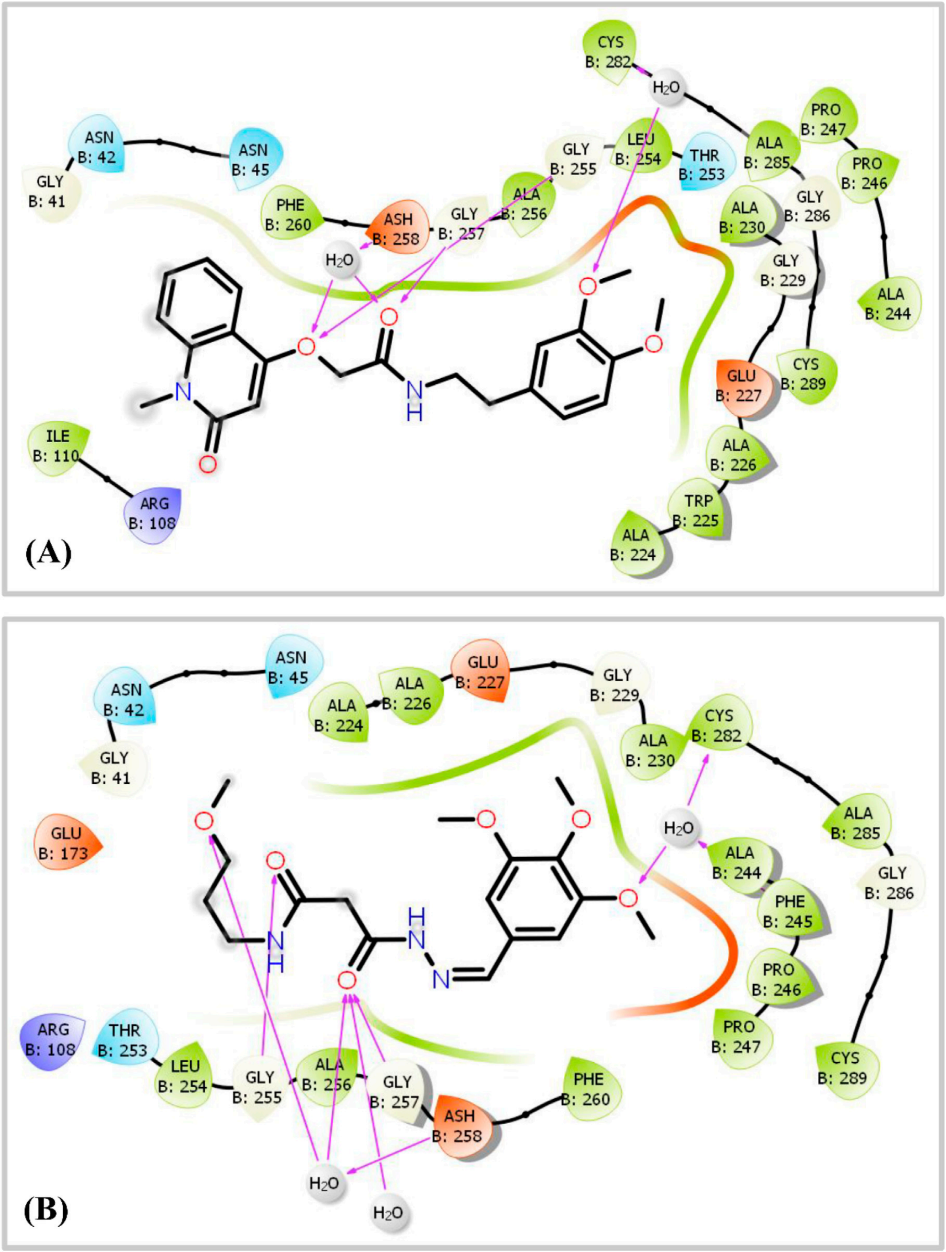
2D Ligand-Protein Interaction Diagram displaying the interactions between two ligands, hit 1 and hit 2, and the KHK-C enzyme (PDB ID: 6W0Z). Panel **(A)** illustrates the binding of hit **1**, where hydrogen bonds (shown as magenta arrows) are formed with specific amino acid residues, identified by their three-letter codes. Panel **(B)** depicts the interaction of hit **2** with the KHK-C enzyme, similarly, highlighting the hydrogen bonds in magenta arrows and detailing the involved amino acid residues.

**FIGURE 9:**
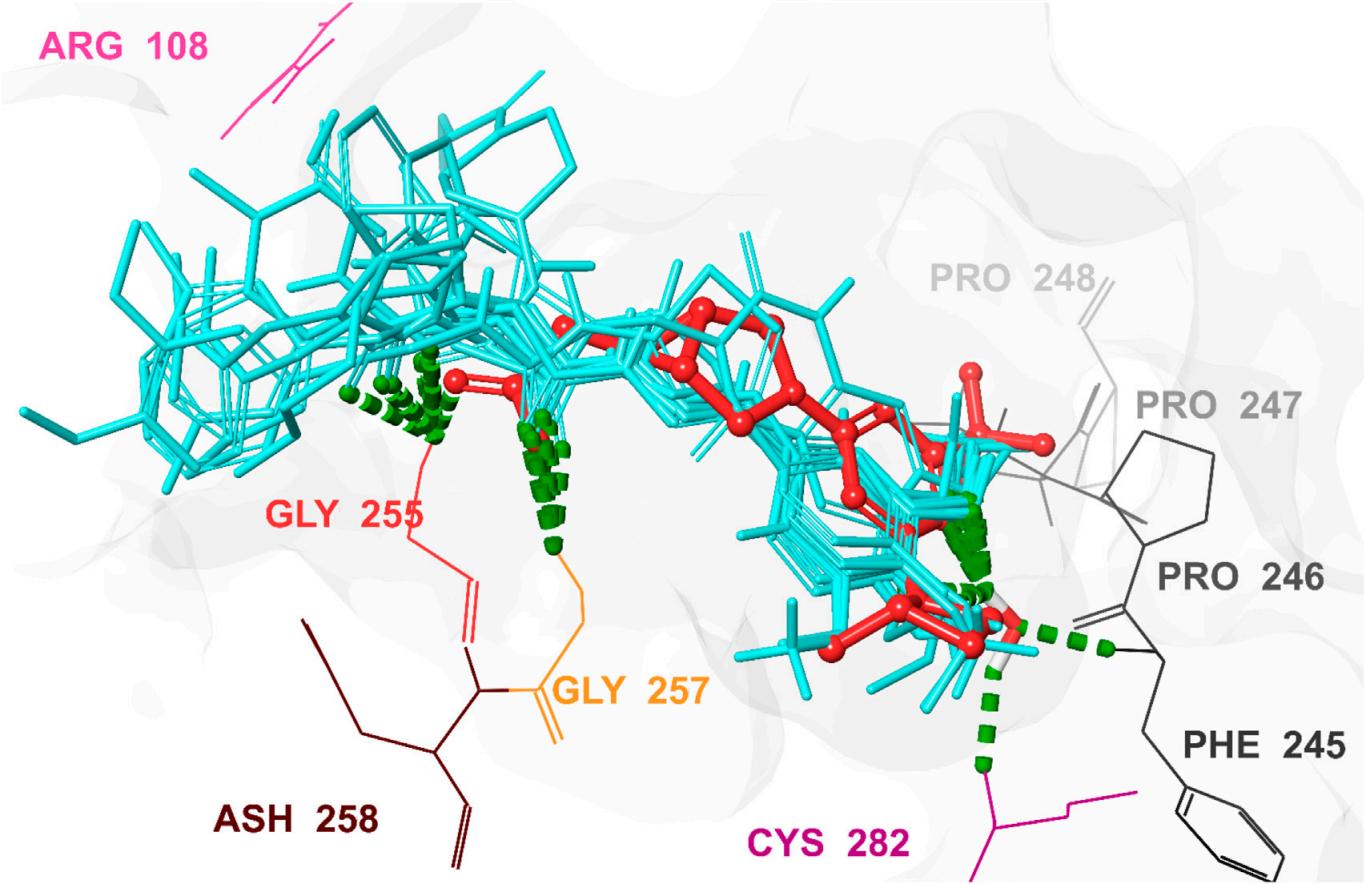
3D interaction pattern of the docked top-ranked hits and the co-crystallized Ligand in the KHK-C binding site (PDB ID: 6W0Z). This figure illustrates the 3D interaction pattern of the docked top-ranked hits and the co-crystallized ligand in the human KHK-C binding site (PDB ID: 6W0Z). Key interacting residues, including Arg108, Phe245, Pro246, Pro247, Pro248, Gly255, Gly257, and Cys282, are highlighted. The co-crystallized ligand is displayed in red, while the superimposed top-ranked hits are shown in cyan, providing a clear comparison of their binding modes and interactions within the active site.

Cross-validation of the molecular docking results was conducted using AutoDock 4.2. All identified hits exhibited more favorable docking scores (−7.4 to −8.5 kcal/mol) compared to the reference ligand PF-06835919 (−7.3 kcal/mol). Hit 4 showed a slightly lower docking score of −7.0 kcal/mol, while the remaining hits consistently outperformed the reference ligand. These results confirm the reliability of the virtual screening protocol in identifying potential KHK-C inhibitors. The interactions of the identified hits with the enzyme binding site were analyzed, with the results presented in Table 1 and Figures 8, 9. The reference ligand occupied the ATP site of KHK-C, forming two hydrogen bonds with Gly255 and Gly257 and a water bridge with ASH258 through its carboxylate group at the solvent-exposed area of the ATP binding site. Additionally, it formed two water bridges with a conserved water molecule, establishing hydrogen bonds with the backbone NH of Phe245 and the backbone CO of Cys282 (Figure 7) via the N3 of the pyrimidine ring. The trifluoromethyl group at C-6 of the pyrimidine ring interacted hydrophobically with the proline loop (Pro245, Pro246, and Pro247), while the 2-methylazetidinyl ring system at C2 occupied the ATP-ribose pocket, interacting hydrophobically with Ala224, Trp225, Ala226, and Phe260. Hits **1–10** exhibited a similar interaction pattern (Table 1; Figure 9) to the reference ligand and previously reported potent KHK-C inhibitors, engaging with key residues in the binding site.

**TABLE 1:** Glide XP docking scores, binding free energy, and molecular interactions of the top 10 ranked hits, the co-crystallized ligand, and the reference inhibitors LY-3522348 and BI-9787 in the KHK-C enzyme binding site.

No	PubChem ID	Docking Score kcal/mol	Binding Energy kcal/mol	Interactions		
				H-Bonds	Water Bridges	Ionic
**Co-crystallized ligand (PF-06835919)**		‒**7.76 ± 0.24**	**‒56.71 ± 0.30**	Gly255 (1.79 Å) Gly257 (2.10 Å)	Phe245 (1.85 and 1.92 Å) ASH258 (1.84 and 2.76 Å) Cys282 (1.85 and 1.91 Å)	-
**LY-3522348**		‒**6.96 ± 0.012**	**‒45.15±0.86**	-	Phe245 (1.92 and 1.92 Å) Cys282 (1.91 and 1.91 Å)	-
**BI-9787**		‒**6.540 ± 0**	**‒28.98 ± 0**	Glu227 (1.92 Å)	Glu227 (1.99 and 2.14 Å)	Glu227 (4.75 Å)
**1**	**6622312**	‒8.23 ± 0.51	‒61.13 ± 0	Gly255 (2.38 Å) Gly257 (1.97 Å)	ASH258 (1.87 and 2.76 Å) (2.72 and 2.76 Å) Cys282 (1.92 and 1.92 Å)	-
**2**	**5341928**	‒9.07 ± 0.15	‒59.61 ± 1.32	Gly255 (1.84 Å) Gly257 (1.84 Å)	Phe245 (1.87 Å and 1.91 Å) ASH258 (2.01 Å and 2.76 Å) and (1.68 Å and 2.76 Å) Cys282 (1.87 Å and 1.92 Å)	-
**3**	**5344383**	‒8.26 ± 0.16	‒61.04 ± 1.17	Gly257 (2.20 Å)	Phe245 (2.09 Å and 1.91 Å) Cys282 (2.09 Å and 1.91 Å) ASH258 (1.86 Å and 2.76 Å)	-
**4**	**2937484**	‒8.69 ± 0.55	‒57.82 ± 1.01	Gly257 (2.16 Å)	Cys282 (1.92 Å and 1.92 Å)	-
**5**	**3239510**	‒9.03 ± 0.02	‒70.69 ± 0.82	Glu227 (2.40 Å) Gly255 (2.46 Å) Gly257 (2.40 Å)	Glu227 (2.02 Å and 2.38 Å) Phe245 (1.99 Å and 1.91 Å) ASH258 (1.67 Å and 2.76 Å) Cys282 (2.25 Å and 1.92 Å)	-
**6**	**3241840**	‒9.10 ± 0.31	‒63.35 ± 0.04	Gly255 (1.99 Å) Gly257 (2.30 Å)	Phe245 (1.91 Å and 1.72 Å) ASH258 (2.49 Å and 2.76 Å) Cys282 (1.92 Å and 1.72 Å)	-
**7**	**1090138**	‒7.79 ± 0.25	‒61.21 ± 0	Thr253 (1.91 Å) Gly255 (2.70 Å) Gly257 (2.36 Å)	Cys282 (1.92 Å and 1.76 Å)	-
**8**	**847629**	‒8.05 ± 0.47	‒57.06 ± 1.6	Gly257 (2.25 Å)	Phe245 (1.96 and 1.91 Å) Cys282 (1.96 and 1.92 Å)	-
**9**	**5307853**	‒8.08 ± 0.08	‒60.52 ± 0.74	Gly255 (2.16 Å) Gly257 (1.90 Å)	PHE245 (2.06 Å and 1.91 Å) Cys282 (2.06 Å and 1.92 Å)	-
**10**	**3239092**	‒8.67 ± 0.34	‒64.39 ± 2.01	Arg108 (2.08 Å) Gly255 (2.17 Å) Gly257 (1.95 Å)	Phe245 (1.71 Å and 1.91 Å) Cys282 (1.71 Å and 1.92 Å)	-

**FIGURE 10:**
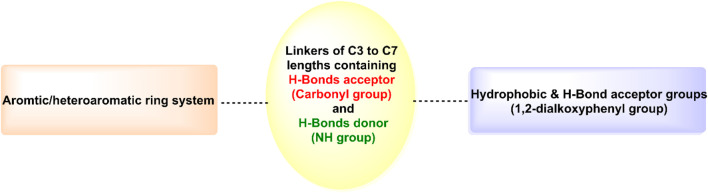
General structural features of the identified hits 1-10.

As shown in Figure 6, hits **1–10** share structural similarities, all containing a 1,2-dialkoxyphenyl group, which plays a crucial role in interactions with the enzyme binding site. This group contributes to four of the five essential pharmacophoric features. Through its oxygen atoms, it forms water bridges with a conserved water molecule, establishing hydrogen bonds with the backbone of Phe245 and Cys282. Additionally, it engages in hydrophobic interactions with the proline loop (PRO246–PRO248) via one of its methyl groups, while the other group interacts similarly with the ATP-ribose pocket, which comprises Ala224, Trp225, Ala226, and Phe260. The identified hits feature C=O and NH functionalities at the center of their structures, actively participating in hydrogen bonding with Glu227, Gly255, Gly257, and Asn258 at the solvent-exposed opening of the binding site. Additionally, these hits incorporate diverse aromatic or heteroaromatic ring systems that extend into the solvent-exposed region, forming interactions with the key residue Arg108 (2.6–2.94 Å), which stabilizes the protein-ligand complex. Superposition of the top-ranked hits within the protein-reference ligand complex of the X-ray crystal structure (Figure 9) reveals that these hits occupy the ATP-binding pocket similarly to the reference ligand, establishing comparable interaction forces. However, unlike the reference ligand, they extend beyond the pocket into the solvent-exposed region, engaging favorably with additional residues. As shown in Figure 10, the identified hits share common structural features, providing insights for the future design and development of novel KHK-C inhibitors for treating fructose-related metabolic diseases.

The identified hits, characterized by planar aromatic residues, effectively occupied the ATP-binding pocket, which is naturally flat and well-suited for their accommodation. These hits formed conserved water-mediated interactions with Phe245, resembling the N1 interaction of the adenine moiety in ATP. Additionally, the triphosphate group of ATP established multiple hydrogen bonds with Gly255, Gly257, and Asp258, a pattern also observed with hits **1–10**. Salt bridges with Arg108 further stabilized ATP within the binding site, ensuring proper orientation for catalysis. Conversely, D-fructose binds within a sterically constrained pocket, where it is stabilized by a bidentate interaction between its HO-3′ and HO-4′ groups with Asp15, along with hydrogen bonds involving HO-1′ and Asn45, HO-4′ and HO-5′ with Gly41 and Asn42, and HO-6′ with Glu29B. These interactions play a crucial role in maintaining fructose in an optimal position for phosphorylation. Among the key catalytic residues, Asp258 functions as a conjugate base, extracting the 1-OH proton from D-fructose to facilitate a 1-O-nucleophilic attack on the γ-phosphate of ATP. Meanwhile, Arg108 serves a dual function by stabilizing ATP’s negatively charged phosphate groups and ensuring their correct alignment for efficient phosphate transfer. Hits **1–10** exhibited strong interactions with these catalytic residues, effectively obstructing the active site and inhibiting fructose phosphorylation. By sterically hindering ATP binding and disrupting the alignment of D-fructose, these hits interfered with the enzyme’s function. Notably, the identified hits, with their extended structures, interacted with both the ATP and fructose-binding sites, forming hydrogen bonds and water bridges with Gly41, Asn42, and Asn45, similar to the interactions observed with fructose. This suggests a dual-inhibition mechanism. As shown in Figures 8, 9, this dual-targeting approach could enhance both potency and specificity, making these hits promising candidates for drug development. By simultaneously impairing substrate recognition and catalytic activity, these inhibitors offer a novel therapeutic strategy for enzyme modulation.

Molecular docking simulation is an important computational tool for identification of ligand’s binding pattern and orientation within a specific macromolecular drug target. However, this tool is not sufficient for ranking multiple ligands based on their experimental affinities. In this regard, a successful structure-based drug design approach should rely on accurate prediction of the energetic basis of ligand-target interactions (El Khoury et al., 2019). To that end, it has been reported that, calculating the ligand free binding affinity to a specific target using molecular mechanics integrated with the generalized Born Surface Area (MM-GBSA) method can significantly improve the accuracy of this prediction (Kaus et al., 2015; El Khoury et al., 2019). Accordingly, the free binding affinity of the top-ranked hits **1–10** towards the KHK-C binding site were computed and the results are provided in Table 2.

**TABLE 2:** Binding free energy of the top-ranked hits and the contribution of individual energy terms to the total binding energy.

Compound No	MMGBSA dG Bind Coulomb	MMGBSA dG Bind Hbond	MMGBSA dG Bind Lipo	MMGBSA dG Bind Packing	MMGBSA dG Bind vdW	MMGBSA dG Bind Covalent	MMGBSA dG Bind Solv GB
Co-crystallized ligand	‒28.26	‒4.22	‒13.31	0.0	‒52.59	1.57	40.09
1	‒34.39	‒3.56	‒15.45	‒0.56	‒47.39	4.18	36.03
2	‒49.68	‒3.78	‒14.02	0	‒4.50	5.08	47.29
3	‒36.19	‒2.69	‒17.54	‒0.02	‒51.58	3.41	43.57
4	‒42.18	‒3.89	‒11.84	0.0	‒36.36	3.34	33.11
5	‒46.73	‒4.56	‒19.42	‒0.25	‒58.87	8.51	50.63
6	‒44.41	‒2.87	‒19.36	‒0.03	‒59.40	8.15	54.58
7	‒31.29	‒3.19	‒12.98	‒1.35	‒49.28	3.77	33.12
8	‒34.41	‒3.25	‒14.43	‒0.23	‒38.55	0.46	33.36
9	‒27.98	‒2.99	‒15.71	0.0	‒54.91	7.25	33.83
10	‒38.32	‒3.71	‒16.83	0.0	‒49.07	4.77	38.78

The bold values represent the docking scores and binding free energy of the reported inhibitors.

The best MM-GBSA energy value was represented by the most negative score (Alameen et al., 2023). While the reference ligand (PF-06835919) had an MM-GBSA energy value of −56.71 kcal/mol, the MM-GBSA values of the top-ranked hits ranged from −57.06 to −70.69 kcal/mol, indicating their relatively high affinity for the KHK-C binding pocket. Among these, the dihydroquinazoline derivative, hit **5**, exhibited the highest binding affinity with an MM-GBSA energy value of −70.69 kcal/mol. To gain insights into the contributions of various interaction forces to the total binding energy of each hit, their ΔG binding values were decomposed into individual components. As shown in Table 2, van der Waals energy (MM-GBSA dG Bind vdW), electrostatic energy (MM-GBSA dG Bind Coulomb), non-polar solvation energy (MM-GBSA dG Bind Lipo), and, to a lesser extent, hydrogen bonding (MM-GBSA dG Hbond) were the major contributors to the strong binding of all hits as well as the reference ligand. In contrast, covalent energy (MM-GBSA dG Bind Covalent) and electrostatic solvation energy (MM-GBSA dG Bind Solv GB) made unfavorable contributions. The results indicate that the identified hits interacted with the KHK-C binding site through forces similar to those of the reference ligand. Notably, hydrophobic interactions, followed by electrostatic interactions, played a key role in the enhanced binding of the top-ranked hits **1–10** and the reference ligand to the target binding pocket. Hits incorporating diethoxy groups attached to the benzene ring hits **5, 6,** and **10** exhibited the highest binding affinities (−70.69, −63.35, and −64.39 kcal/mol, respectively). These hits interacted with two hydrophobic loops (Pro246, Pro247, and Pro248) as well as another loop (Ala224, Trp225, Ala226, and Phe260), further supporting the critical role of hydrophobic interactions in strong binding.

A comparative study of binding free energy was conducted to assess the interaction of the identified hits **1–10** with the target relative to reported inhibitors (Table 1). The identified hits exhibited stronger binding energies compared to PF-06835919 (−56.71 kcal/mol). Hits such as hit **1** (−61.13 kcal/mol), hit **3** (−61.04 kcal/mol), and particularly hit **5** (−70.69 kcal/mol) showed more favorable binding energies, indicating a stronger affinity for the target. Hit **5** stood out with the lowest binding energy, suggesting tighter binding than PF-06835919. Hits **6** (−63.35 kcal/mol) and **9** (−60.52 kcal/mol) also exhibited stronger binding than PF-06835919, implying superior binding affinity with the target. The IC_50_ values for the reported inhibitors (Figure 2) were 0.01 μM for PF-06835919, 0.02 μM for LY-3522348, and 0.012 μM for BI-9787. Although the IC_50_ values for the identified hits **1–10** had not been experimentally determined, their stronger binding energies suggest they could demonstrate similar or enhanced potency compared to the reported inhibitors. LY-3522348 (−45.15 kcal/mol) and BI-9787 (−28.98 kcal/mol) exhibited weaker binding affinities than the identified hits. These findings support the potential of the identified hits as more effective inhibitors with potentially higher potency and stronger target affinity than the current reported inhibitors.

### 3.3 ADMET profiling

The top-ranked hits **1–10** exhibited acceptable ADME-compliance scores (Supplementary Table S1), indicating that their property descriptor values fell within the permissible range for approved drugs. The calculated oral bioavailability of these hits ranged from 80% to 100%, surpassing BI-9787’s bioavailability of 68% in rats and comparable to or better than LY-3522348 (87%) and PF-06835919 (95% in rats). Hits **1, 3, 6,** and **7** showed moderate blood-brain barrier (BBB) penetration (QPlogBB values from −1 to 0), while the remaining hits exhibited low BBB penetration (QPlogBB values <−1). This suggests that the identified hits are more likely to be specific to their hepatic target, minimizing the risk of neurological side effects.

The permeability profiles of hits **1–10** indicated superior membrane permeability, with predicted MDCK cell permeability values ranging from 113 nm/s to 2,242 nm/s, compared to LY-3522348 (4.4%) and BI-9787 (low permeability). The hits exhibited a higher permeability, suggesting enhanced intestinal absorption, improved oral bioavailability, and potential for better systemic uptake.

The hits **1–10** demonstrated weak binding to human serum albumin, as indicated by their low QPlogKhsa values, suggesting a higher free drug fraction. This can potentially enhance their therapeutic efficacy by improving distribution and target engagement. In contrast, BI-9787 has more than 99% plasma protein binding, and LY-3522348 is 85% bound. Lower plasma protein binding may allow for greater systemic exposure and a more rapid onset of action, reducing the risk of off-target interactions associated with high plasma protein binding.

The number of predicted metabolic reactions for hits **1–10** ranged from 4 to 7, indicating moderate metabolism in the liver. This suggests that the compounds may reach their site of action in their intact forms. However, their metabolic stability is slightly lower than LY-3522348, which is predicted to undergo only 1 metabolic reaction. The higher metabolic stability of LY-3522348 may need to be considered for optimization in the identified hits.

For the assessment of cardiotoxicity, hits **1, 2, 4–6** exhibited predicted IC_50_ values above −5 for hERG inhibition, suggesting a lower likelihood of cardiotoxicity. In contrast, hits **3, 7–10** exhibited higher IC_50_ values (>–5), indicating potential cardiotoxicity and were excluded from further investigations. LY-3522348 also exhibited a minimal risk of cardiac toxicity, with an IC_50_ value of −4.6, further confirming its favorable safety profile. The identified hits may require further optimization to reduce the risk of cardiotoxicity.

**FIGURE 11:**
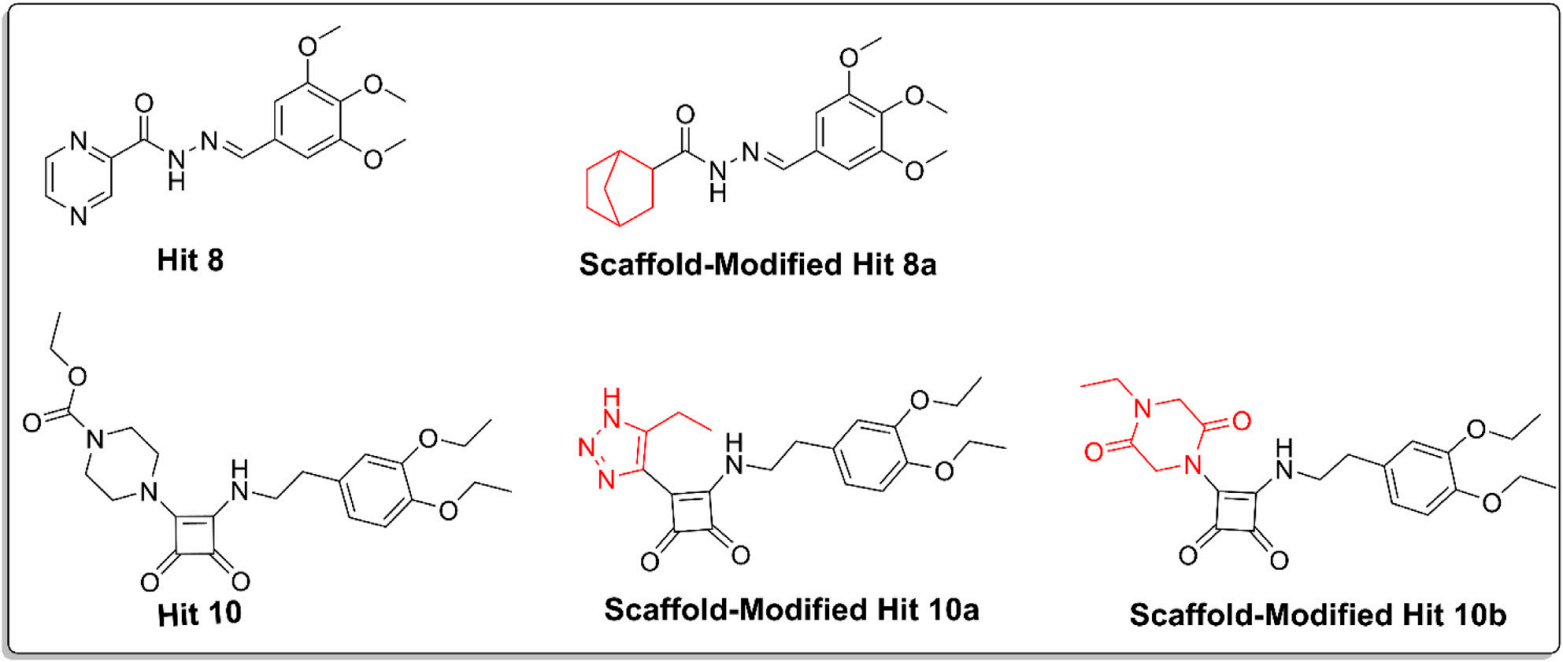
Chemical structures of hit 10, scaffold-modified hit10a, scaffold-modified hit 10b, hit 8, and scaffold-modified hit8a, illustrating the structural modifications made to reduce cardiotoxicity. The replaceable groups, highlighted in red, indicate the modifications.

Scaffold replacement was performed on the excluded hits **3, 7–10** to reduce cardiotoxicity while maintaining their pharmacological potential. Ten replacements were conducted for each excluded hit, focusing on reducing lipophilicity, decreasing planarity, minimizing basicity, and enhancing hydrogen bonding. Among these, three analogs (Figure 11) demonstrated significant reduction in cardiotoxicity.

For hit 8, the replacement of the pyrazine ring with a bicyclo [2.2.1]heptane structure resulted in hit 8a, which showed a docking score of −8.01 kcal/mol and successfully reduced cardiotoxicity to the specified limit. For hit 10, the first analog (**hit 10a**) involved replacing piperazine with 1,2,3-triazole, yielding a docking score of −7.8 kcal/mol, which was still higher than the co-crystal ligand. This modification reduced the basicity and planarity, minimizing hERG channel interactions. The second analog for hit **10 (hit 10b)** replaced piperazine with 2,5-diketopiperazine, achieving a docking score of −8.4 kcal/mol. This modification reduced basicity and increased polarity, leading to reduced lipophilicity and improved solubility. The reduction in cardiotoxicity for these modifications makes them promising candidates for further evaluation.

In general, the outcome of this analysis indicates that hits **1,2** and **4-6** displayed acceptable drug-likeness, pharmacokinetics features, and safety profiles, thereby denoting their potential as KHK-C inhibitors for future treatment of fructose metabolic diseases.

### 3.4 MD simulations

**FIGURE 12:**
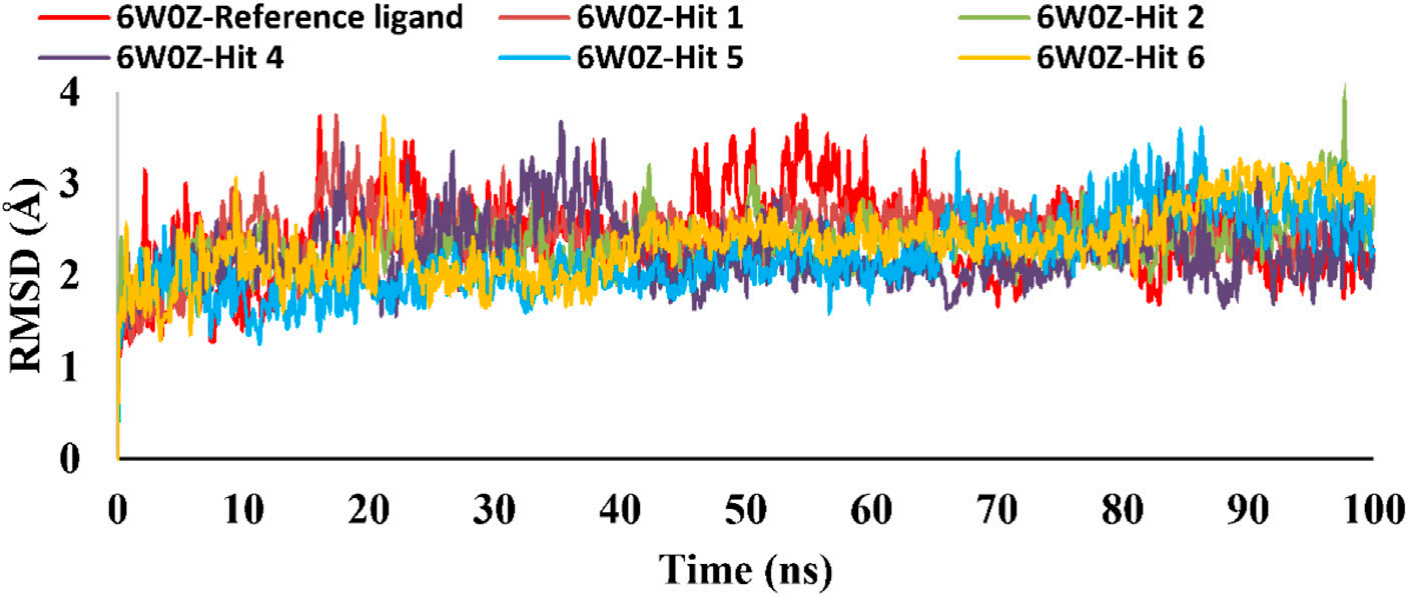
RMSD plot for the simulated docked complexes of the top-ranked hits 1,2, and 4-6 as well as the reference ligand during 100 ns MD simulations. The X-axis represents the time in ns and the Y-axis represents the RMSD value of each complex in Å.

**FIGURE 13:**
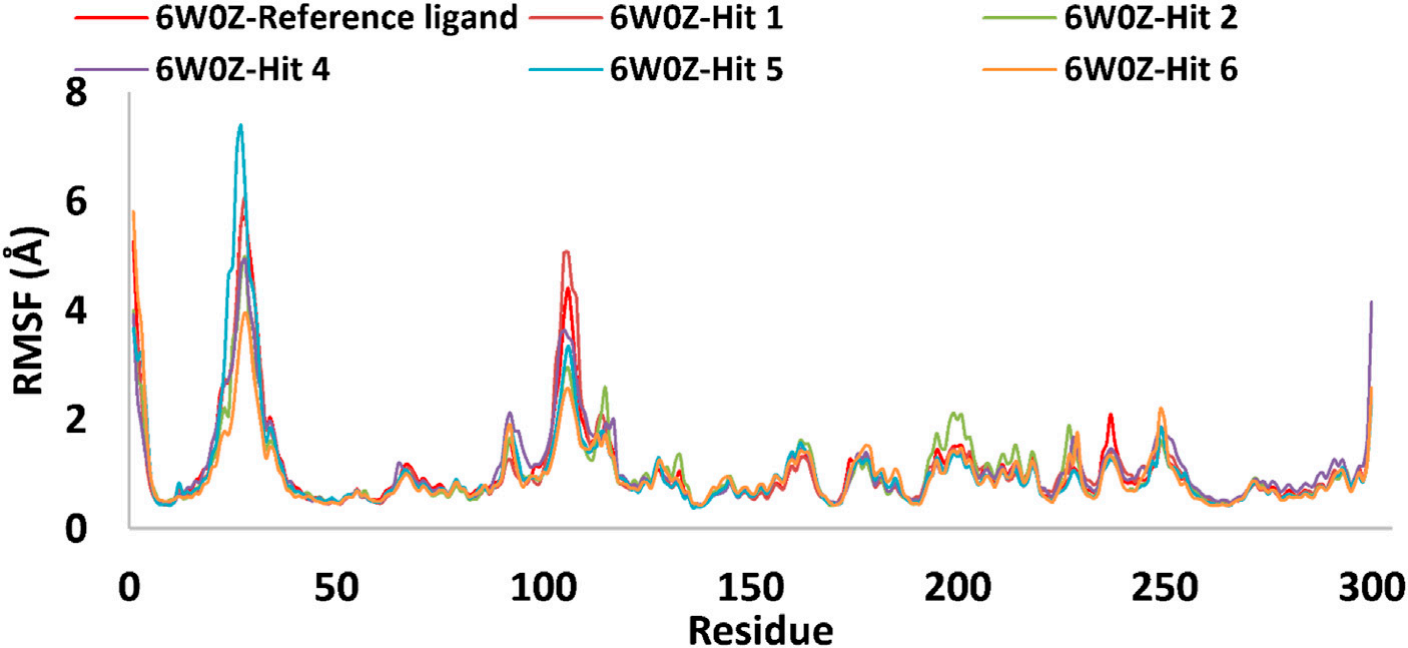
RMSF plot for the simulated docked complexes of the top-ranked hits 1,2, and 4-6 as well as the reference ligand during 100 ns MD simulations. The X-axis displayed the number of the target’s residues, while the Y-axis showed the RMSF value of each simulated docked complex.

**FIGURE 14:**
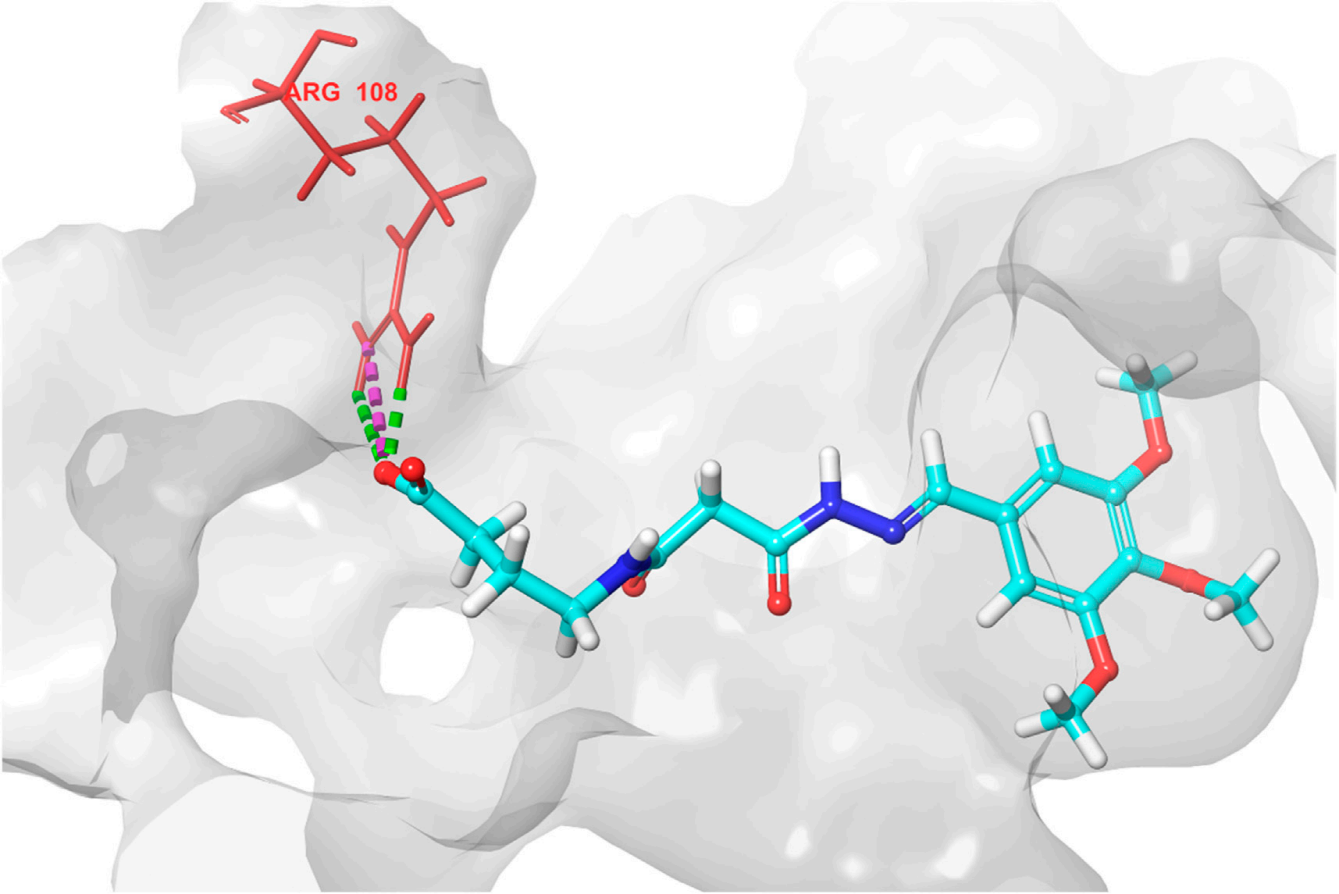
3D interaction diagram of the designed hit 2 analog, showing the ionic interaction and two hydrogen bonds between the newly introduced carboxylate group and Arg108.

Based on the predicted ADMET properties, hits **1, 2**, and **4-6** were selected for 100 ns MD simulation. The stability of the best-docked conformations of the hits and the reference ligand (PF-06835919) were monitored in an aqueous system using the Desmond package. The RMSD values for the protein C-α atoms relative to the initial structure were used to evaluate complex stability. The reference ligand exhibited an average RMSD of 2.41 ± 0.43 Å, with unstable fluctuations throughout the simulation (Table 3; Figure 12). For hits **1** and **2**, the average RMSD values were 2.47 ± 0.31 Å and 2.34 ± 0.27 Å, respectively, with initial instability for the first 15 ns before stabilizing. The RMSD of the hit **4** complex was 2.24 ± 0.34 Å, showing drift up to 40 ns, after which it stabilized. Hit **5** reached equilibrium quickly (average RMSD = 2.19 ± 0.41 Å) but exhibited higher deviations after 65 ns? Hit **6** showed an average RMSD of 2.36 ± 0.38 Å, reaching a steady state around 23 ns? Overall, the structures of KHK-C remained stable after complexation with hits **1, 2**, and **4-6**, with RMSD profiles better than the reference ligand, indicating their potential as KHK-C inhibitors. The RMSF analysis (Figure 13) showed that the protein residues fluctuated mainly in two regions (>3 Å): Lys22-Arg31 and Asn102-Asn107, which are not critical for enzyme inhibition. Critical residues in the ATP binding site (Phe245, Pro246, Pro247, Thr253, Gly255, Gly257, Phe260, and Cys282) displayed limited mobility (RMSD <1.5 Å) during the simulation. Hits **2**, **5**, and **6** enhanced the stability of Arg108 compared to the reference ligand. Protein-ligand interaction analysis was performed to examine intermolecular forces. As shown in Supplementary Figures S4–S9, different types of non-bonding interaction forces were involved in the ligand–protein complex overall stability. The reference ligand showed stable Water Bridge interactions with critical residues Phe245 and Cys282, and moderate hydrophobic interactions with Ala226 and Phe260 (Supplementary Figure S4). For hit 1 (Supplementary Figure S5), dimethoxy groups formed multiple Water Bridges with Cys282 and Phe245, while the amide carbonyl group interacted with Thr253, Gly255, Ala256, and Gly257. These interactions were weaker than those of the reference ligand, indicating lower KHK-C inhibitory potential for hit **1**. Hit **2** (Supplementary Figure S6) retained the non-bonding interactions observed in molecular docking and formed new stable Water Bridges with Phe245 and Cys282. It also formed H-Bonds with key residues like Glu227, Gly255, Gly257, and ASH258. The interaction fraction was greater than 1, suggesting stronger interactions compared to the reference ligand. A new moderately stable H-Bond was observed with Thr253, which persisted for 40% of the simulation time. However, the interaction with Arg108 was minimal compared to the reference ligand. To improve binding affinity, an analog of hit **2** was designed by introducing a free carboxylate group at the terminal side chain to form an ionic bond with Arg108 (Figure 14). Glide XP docking of this analog resulted in an improved docking score (−9.23 kcal/mol) and the formation of an ionic bond with Arg108, alongside two H-Bonds.

**TABLE 3:** C-α RMSD values, including minimum, maximum, and average for the top-ranked hits 1, 2 and 4-6 complexes with KHK-C (PDB ID: 6W0Z). This also includes comparative data for the co-crystallized ligand complex.

Protein	Maximum	Minimum	Average
6W0Z-Co-crystallized ligand	3.72	1.12	2.41
6W0Z-Hit **1**	3.73	1.11	2.47
6W0Z-Hit **2**	3.90	1.40	2.34
6W0Z-Hit **4**	3.67	1.21	2.24
6W0Z-Hit **5**	3.58	1.02	2.19
6W0Z-Hit **6**	3.70	1.29	2.36

**FIGURE 15:**
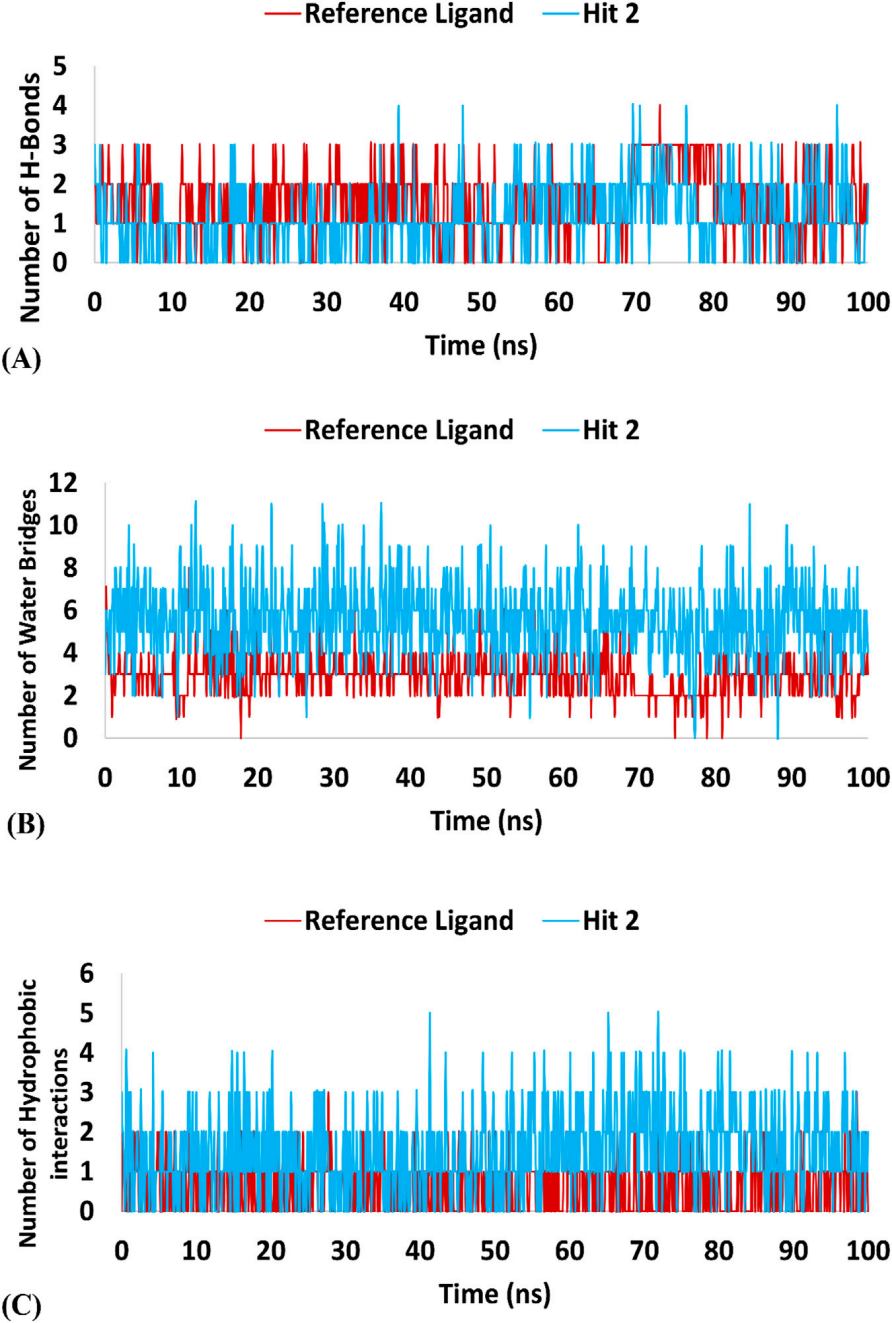
Interaction forces established during the 100 ns MD Simulation of hit 2 and the reference ligand with the KHK-C Binding Site (PDB: 6W0Z). Panel **(A)** H-Bonds: Displays the hydrogen bond interactions formed during the simulation between hit 2 (and the reference ligand) and the KHK-C binding site. Panel **(B)** Water Bridges: illustrates the number of water bridges formed between the ligands and the binding site throughout the MD simulation. Panel **(C)** Hydrophobic Interactions: Shows the hydrophobic interactions established during the simulation, indicating their contribution to ligand stability within the binding site.

Regarding hits **4–6** (Supplementary Figures 7–9), they demonstrated relatively weak binding interactions during MD simulations with the key residues Phe245 and Cys282 (hits **5** and **6**), or Gly255 and Gly257 (hit **4**), compared to the reference ligand, suggesting their lower potential for KHK-C inhibition. Hit **2** interacted with the most critical residues in the enzyme binding pocket and created intermolecular bonds that were stronger than those made by the reference ligand. Moreover, hit 2 did not engage in binding interactions with residues conserved in KHK-A (Asp15, Asn42, Asn45, Arg141, and Lys174), indicating its potential selectivity for targeting KHK-C. In terms of the number of interactions, hit **2** formed similar numbers of H-Bonds with the enzyme’s binding site residues like the reference ligand (averaging 1.15 and 1.52, respectively), but the number of Water Bridges established by hit **2** were nearly twice those formed by the reference ligand, averaging 5.5 and 2.9, respectively (Table 4; Figures 15A, B). Additionally, hit 2 exhibited a higher number of hydrophobic contacts compared to the reference ligand (averaging 1.47 and 0.64, respectively) (Table 4; Figure 15C). These results indicate that hit **2** demonstrated more stable interactions compared to the reference ligand, highlighting its potential as a potent KHK-C inhibitor worthy of experimental validation.

**TABLE 4:** Maximum, minimum, and average counts of H-Bonds, Water Bridges and hydrophobic interactions formed during a 100 ns MD simulation of Hit 2 and the reference ligand complexes with KHK-C (PDB: 6W0Z).

Molecule	Number of H-Bonds			Number of Water Bridges			Number of hydrophobic interactions		
	Maximum	Minimum	Average	Maximum	Minimum	Average	Maximum	Minimum	Average
Reference ligand	4	1	1.52	8	1	2.9	3	1	0.64
Hit 2	4	1	1.15	11	1	5.5	5	1	1.47

**FIGURE 16:**
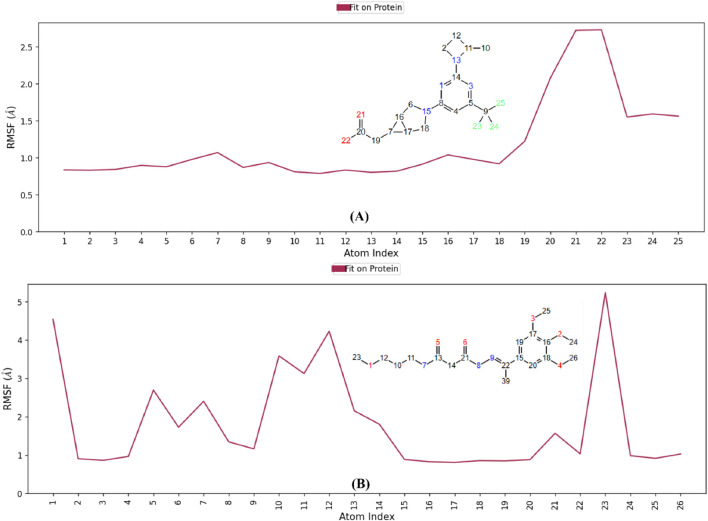
RMSF analysis of ligand dynamics in the KHK-C binding pocket. **(A)** Co-crystallized ligand **(B)** Hit **2**.

The RMSF analysis of the co-crystal ligand and hit **2** (Figure 16) shows fluctuations in key atoms, providing insights into their stability and flexibility within the KHK-C binding pocket. For the co-crystal ligand, the carboxylate oxygens showed high fluctuations (∼2.73 Å) (Figure 16A), indicating dynamic behavior despite forming strong interactions with Gly255, Gly257, and Arg108. The CF_3_ group fluorines exhibited fluctuations (∼1.2–2.3 Å), suggesting conformational shifts in the proline loop (Pro246–Pro248). Hydrogen bond occupancy analysis revealed intermittent interactions with Gly255, Gly257, and Arg108 (Supplementary Figure S4), with occupancy below 20%, and weak water-bridge interactions with Asn58. In contrast, hit 2 showed lower RMSF values, indicating greater stability within the binding pocket. Atoms 2 and 3 (oxygens) (Figure 16B) showed limited fluctuations (below 1 Å) and participated in stable water-bridge interactions with Cys282 and Phe245, with hydrogen bond occupancy above 95% (Supplementary Figure S6). Methyl groups 24, 25, and 26 interacted with the hydrophobic pocket formed by Pro246–Pro248, showing minimal fluctuations (below 1.2 Å). Atom 5 (oxygen) showed a high RMSF of 2.6 Å, with intermittent interactions with Thr253, while atom 8 (nitrogen) exhibited a stable interaction with Glu227 (RMSF of 1.3 Å). The terminal methoxy group (atoms 1 and 23) displayed the highest flexibility, with fluctuations exceeding 4 Å. Overall, the water-bridge and hydrophobic interactions of hit **2** contributed to a more stable binding mode, with minimal fluctuations observed in key atoms, resulting in a stronger and more stable binding within the KHK-C binding site. These findings support hit 2’s potential as a promising KHK-C inhibitor.

**FIGURE 17:**
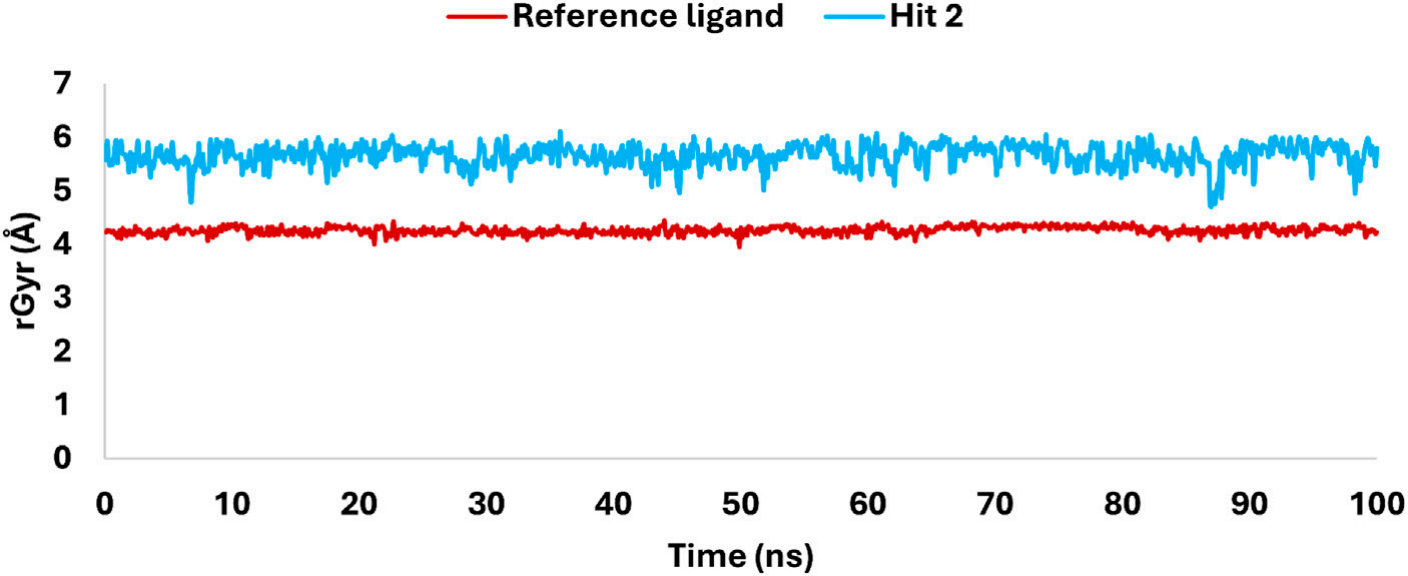
Variations in rGyr of hit 2 and the reference ligand, highlighting flexibility and structural rigidity within the KHK-C catalytic site.

The radius of gyration (rGyr) analysis over a 100 ns simulation (Figure 17) revealed key insights into the structural stability and flexibility of the ligands within the KHK-C binding site. The reference ligand maintained a compact conformation with an average rGyr of 4 Å, indicating stable binding with minimal fluctuations. In contrast, hit **2** exhibited a higher rGyr, fluctuating between 5.5 and 6 Å, suggesting a more extended conformation and greater flexibility within the binding pocket. This flexibility likely allowed dynamic interactions with key catalytic residues, enhancing its binding potential. Both ligands showed overall structural stability, with no significant deviations or drastic conformational changes observed during the simulation. The differences in rGyr between the ligands indicated that hit **2**’s flexibility might enable the exploration of additional binding modes, potentially optimizing its interactions with critical residues. This flexibility, facilitated by multiple rotatable bonds, contrasts with the reference ligand’s rigid multicyclic structure. The analysis supports that hit **2** maintained its integrity within the KHK-C catalytic site, suggesting its potential for further investigation as a promising inhibitor.

In KHK-C, hit **2** demonstrated a significantly higher binding affinity than PF-06835919, with a docking score of −9.07 and a binding affinity of −59.61 kcal/mol, compared to PF-06835919s docking score of −7.76 and binding affinity of −56.71 kcal/mol. Hit 2 also exhibited dynamically stable behavior, forming a more stable complex and establishing stronger interactions with critical residues, both in type and intensity, compared to PF-06835919. For KHK-A, docking studies using the crystal structure (PDB ID: **8OME**) revealed that hit **2** displayed a stronger binding affinity, with a binding free energy of −56.8 kcal/mol and a docking score of −6.79, surpassing PF-06835919, which had a binding affinity of −3.49 kcal/mol and a docking score of −4.36. These results suggest that hit **2** exhibited superior binding affinity and stability, interacting effectively with key residues in both KHK-C and KHK-A, making it a promising candidate for dual inhibition of both isoforms.

## 4 Discussion

Identification of new lead molecules using Computer Aided Drug Design (CADD) is considered one of the major approaches of contemporary pre-clinical drug discovery process (Prajisha et al., 2022). CADD typically involves the use of various computational techniques and software programs, often in combination, to search for new drug candidates. Over the last 2 decades, CADD has had a significant impact on pre-clinical drug development, and its use continues to expand as virtual screening and molecular docking technologies evolve. As a result, several clinically useful drugs have been developed with the aid of CADD during this period (Sabe et al., 2021). Accordingly, the present study employed multiple computational methods, such as pharmacophore modeling, molecular docking, MM-GBSA, prediction, ADME-T profiling, and MD simulation (as shown in Figure 3), to explore the NCI small molecule library for novel KHK-C isoform inhibitors. These inhibitors are expected to play a crucial role in the future treatment of metabolic diseases associated with excessive fructose intake, including NIDDM, atherogenic dyslipidemia, NAFLD, NASH, and obesity. Particularly, obesity is considered a major health problem due to its substantial contribution to increasing the risk of several disorders, such as myocardial infarction, stroke, and various types of cancer (Bluher, 2019). Thus far, the currently available prevention and treatment strategies have not resulted in significant improvement, and the global prevalence of this condition continues to rise. Additionally, the limitations of effective obesity treatments often necessitate bariatric surgery as a last resort. However, it is evident that bariatric surgery cannot address the global increase in obesity prevalence (Busebee et al., 2023) and it may cause both early and late complications (Dahan et al., 2024). The former includes infection, an elevated risk of blood clots, anastomotic leaks, and bleeding at the surgical site, which may necessitate blood transfusion (Vitiello et al., 2021; Golzarand et al., 2022; Ab Majid et al., 2024). The latter includes the formation of gallstones due to rapid weight loss, nutritional deficiencies from reduced food intake and absorption, and weight regain owing to various factors, such as stretching of the stomach, poor dietary habits, hernias particularly at the site of the surgical incision and ulcers that may develop at the connection sites in the gastrointestinal tract (Valera et al., 2023; Ab Majid et al., 2024; Wang et al., 2024; Yakout et al., 2024). Therefore, discovering new medications targeting KHK-C may be more effective in reducing excess weight for individuals with severe obesity than relying on bariatric surgical intervention.

Pharmacophore modeling is most commonly used in virtual screening to identify compounds that demonstrate the desired pharmacological action (Voet et al., 2014). In the present study, the X-ray crystal structure of KHK-C (PDB: **6W0Z**) in complex with compound PF-06835919 (IC_50_ value 0.01 µM) was retrieved from the Protein Data Bank. The protein crystal structure has a resolution of 2.30 Å. In this study, we performed hypothesis validation to evaluate the generated hypothesis for identifying potential KHK-C inhibitors. This validation was conducted using a dataset of experimentally validated compounds, which were sourced from literature (Maryanoff et al., 2011; Huard et al., 2017; Durham et al., 2023; Zhu et al., 2023; Heine et al., 2024), as actives and decoys. The dataset included 45 ligands, consisting of 30 actives and 15 decoys. The active compounds were carefully ranked according to their binding affinities, and the overall validation process was designed to rigorously assess the ability of the hypothesis to predict active compounds. Key metrics of hypothesis validation showed promising results. The ROC value was 0.81 (Supplementary Figure S1), demonstrating good classification performance between actives and decoys. Additionally, the RIE (Ranking Information Entropy) was 1.50, indicating a well-ordered ranking where actives were placed significantly higher than the decoys. The AUC stood at 0.62, a robust indication of the hypothesis’s ability to accumulate actives effectively over time. The BEDROC values for different alpha values (160.9, 20.0, and 8.0) were 1.000, 1.000, and 0.977, respectively, further affirming the hypothesis’s strength in identifying actives early in the ranking list. This high BEDROC value reflects the model’s capability to capture the relevant active compounds with high precision in the top-ranked positions. When analyzing the results of the screening (Supplementary Figure S1), 56.7% of the actives were positioned in the top 20% of the ranked decoys. This suggests a significant enrichment, supporting the idea that the hypothesis is proficient in distinguishing between active and inactive compounds. The EF for various sample sizes, such as 1%, 2%, and 5%, were consistently high, with EF values of 1.5 and EF and EF'* values showing favorable outcomes, especially for higher percentages of actives recovered. The hit rate at the top 50 ligands was 52%, demonstrating a high success rate for recovering active compounds from the top ranks. Furthermore, the validation reported an average number of outranking decoys of 1, indicating a solid performance in ensuring that active compounds were ranked above the majority of decoys. The Enrichment Factor (EF') for recovering 50% of actives was 7.5, with EF values showing even higher enrichment as more actives were considered, demonstrating strong prediction power in identifying relevant compounds. Overall, the validation results confirm that the hypothesis is highly effective for identifying potential KHK-C inhibitors. The consistent and high enrichment factors, robust ROC scores, and favorable BEDROC values, combined with the high hit rate in the top-ranked compounds, demonstrate the validity of the hypothesis as a reliable method for discovering KHK-C inhibitors. These findings provide strong support for advancing the hypothesis as a foundational tool for further *in silico* studies and experimental validation in KHK-C inhibitor research.

The accurate assignment of feature requirements and permissiveness in a pharmacophore model is crucial for optimizing the prediction of ligand-target interactions, ensuring that the model can efficiently guide the design of novel compounds with potent binding affinity and specificity. In this context, the ring feature and the two hydrophobic features appeared in all the retrieved active compounds, highlighting their crucial role in ligand binding and interaction with the target. This consistent presence across all active compounds strongly suggested that these features were essential for achieving binding affinity and specificity. As a result, these features were assigned as required in the pharmacophore model, ensuring that any ligand that did not meet these criteria would likely fail to interact effectively with the target. On the other hand, the negative center appeared in 14 out of the 26 retrieved actives, which corresponded to approximately 53.8% of the actives. While not as universally present as the required features, its frequent occurrence in more than half of the active compounds indicated its significant role in enhancing binding and stability. Thus, this feature was assigned as permitted, meaning that ligands could still be considered active even if they did not include a negative center, but its presence provided an added advantage in interactions. Similarly, the hydrogen bond acceptor feature appeared in 13 out of the 26 retrieved actives, accounting for 50% of the actives. This showed that while the hydrogen bond acceptor feature was not absolutely required, it was still an important component for a substantial portion of the active ligands. Therefore, it was also assigned as permitted, allowing flexibility in the pharmacophore model, where the feature could be present in some active compounds but was not mandatory for binding. Overall, the ring and hydrophobic features were considered essential for ligand binding and assigned as required, while the negative center and hydrogen bond acceptor features were observed in a significant portion of the actives and were therefore assigned as permitted. This allowed for a flexible pharmacophore model that could accommodate slight variations in ligand structure while still predicting potent binding interactions with the target.

The generated pharmacophore model was then employed to screen the prepared NCI library. This selection process resulted in a total of 9,000 molecules being chosen for structure-based virtual screening. The compounds were ranked according to their phase fitness scores, which were utilized to assess their potential as active inhibitors. The selected molecules were then analyzed for their ability to interact effectively with the target, aiming to identify the most promising candidates for further investigation and development.

Molecular docking has become a crucial component of the *in silico* drug design process, as it predicts protein-ligand interactions at the molecular level (Agu et al., 2023). In this study, the number of molecules obtained from pharmacophore-based virtual screening (over 9,000) was too large to be efficiently processed using Glide extra precision (XP) docking. Therefore, high-throughput virtual screening (HTVS) was initially employed, reducing the number of compounds to 700. Subsequent docking using the standard precision (SP) mode yielded 37 compounds with docking scores better than that of the reference ligand. These 37 compounds were then subjected to Glide XP docking, identifying 10 hits with docking scores ranging from −7.79 to −9.10 kcal/mol, all of which outperformed the co-crystallized ligand (−7.76 kcal/mol). Among the top-ranked hits, hit **6** exhibited the highest docking score (−9.10 kcal/mol). The chemical structures of the top-ranked hits are provided in Figure 6. The docking protocol was validated by re-docking the co-crystallized ligand into the same binding pocket. The Root Mean Square Deviation deviation (RMSD) between the docked conformation and the native ligand was low (0.245 Å) (Figure 7), confirming the reliability of the docking protocol used in this study.

Further, to validate the virtual screening workflow, we conducted a rigorous enrichment analysis using a dataset consisting the top 10 hits identified in this study as actives and 18 inactives sourced from the literature (Maryanoff et al., 2011; Le et al., 2016; Huard et al., 2017; Zhu et al., 2023). The chemical structures of the inactives are provided in Supplementary Figure S2, along with the ROC curve and % screen figure for visual reference. The inactives were selected based on their reported IC_50_ values (>1 µM against KHK-C), ensuring a robust dataset for evaluating the screening model’s performance. Further, the enrichment analysis demonstrated exceptional virtual screening performance, achieving a perfect ROC score of 1.0, as depicted in the ROC curve in Supplementary Figure S3. This indicates flawless discrimination between actives and inactives. Additionally, BEDROC values of 1.0 confirmed optimal early recognition of actives, highlighting the model’s ability to prioritize true hits at the top of the ranked list. The relative initial enrichment (RIE) value of 4.54 further validated the strong separation between actives and inactives, underscoring the model’s precision. The area under the accumulation curve (0.89) reflected high ranking efficiency, demonstrating that the actives were consistently placed in the top ranks. Enrichment factors (EF = 4.6) remained stable across all sample sizes, indicating the model’s reliability and scalability. Notably, 100% of the actives appeared within the top 20% of the ranked results, as illustrated in the % screen figure in Supplementary Figure S3, with no actives outranked by inactives. This precise prioritization ensures that true hits are efficiently identified without false positives. The actives were well-distributed within the top positions, further reinforcing the model’s effectiveness. The consistent performance across multiple metrics ROC, BEDROC, RIE, and EF validates the robustness of the virtual screening workflow. These results confirm the model’s ability to accurately identify potential KHK-C inhibitors, making it a reliable tool for drug discovery efforts.

The docking program AutoDock 4.2 was employed to perform cross-validation of the molecular docking results. The outcomes demonstrated that all identified hits exhibited more favorable docking scores (−7.4 to −8.5 kcal/mol) compared to the reference ligand PF-06835919, which showed a binding affinity of −7.3 kcal/mol. Notably, hit 4 displayed a slightly lower docking score of −7.0 kcal/mol, while the remaining hits consistently outperformed the reference ligand. These findings further reinforce the reliability of the virtual screening protocol and validate the potential of the identified hits as promising KHK-C inhibitors. Next, the interactions of these hits with the enzyme binding site were analyzed and the results are indicated in Table 1 as well as Figures 8, 9. It has been shown that the reference ligand occupied the ATP site of KHK-C (Zhu et al., 2023). It displayed two H-Bonds with Gly255 and Gly257 and one Water Bridge with ASH258 by its carboxylate group at the solvent exposed area of the ATP binding site. Moreover, it created additional two Water Bridges with a conserved water molecule forming H-Bonds with the backbone NH of Phe245, and the backbone CO of Cys282 (Figure 7), by the N3 of the pyrimidine ring. Furthermore, the trifluromethyl group attached to C-6 of the pyrimidine ring was within the range of the hydrophobic interactions with the proline loop (Pro245, Pro246 and Pro247). On the other hand, the 2-methylazetidinyl ring system attached to C2 of the pyrimidine ring filled the ATP-ribose pocket and exhibited hydrophobic interactions with Ala224, Trp225, Ala226 and Phe260. Hits **1–10** demonstrated an interaction pattern (Table 1; Figure 9) similar to that of the reference ligand and the previously reported potent KHK-C inhibitors, interacting with the critical residues of the binding site (Maryanoff et al., 2011; Huard et al., 2017; Futatsugi et al., 2020; Zhu et al., 2023). As shown in Figure 6, hits **1–10** share structural similarities, with all containing a 1,2-dialkoxyphenyl group that plays a crucial role in interactions with the enzyme binding site. This group contributes to four out of five essential pharmacophoric features. Through its oxygen atoms, it forms water bridges with a conserved water molecule, establishing hydrogen bonds with the backbone of Phe245 and Cys282. Additionally, it engages in hydrophobic interactions with the proline loop (PRO246–PRO248) via one of its methyl groups. Meanwhile, the other group interacts similarly with the ATP-ribose pocket, which comprises Ala224, Trp225, Ala226, and Phe260. Notably, the identified hits feature C=O and NH functionalities at the center of their structures, which actively participate in hydrogen bonding with Glu227, Gly255, Gly257, and Asn258 at the solvent-exposed opening of the binding site. Moreover, these hits incorporate diverse aromatic or heteroaromatic ring systems that extend into the solvent-exposed region, forming interactions with the key residue Arg108 (2.6–2.94 Å), a stabilizing factor for the protein-ligand complex (Chan et al., 2010). Superposition of the top-ranked hits within the protein-reference ligand complex of the X-ray crystal structure (Figure 9) reveals that these hits occupy the ATP-binding pocket similarly to the reference ligand, establishing comparable interaction forces. However, unlike the reference ligand, they extend beyond the pocket into the solvent-exposed region, engaging favorably with additional residues. As shown in Figure 10, the identified hits share common structural features, offering valuable insights into the future design and development of novel KHK-C inhibitors for treating fructose-related metabolic diseases. The identified hits, characterized by planar aromatic residues, effectively occupied the ATP-binding pocket, which is naturally flat and well-suited for their accommodation. These hits formed conserved water-mediated interactions with Phe245, resembling the N1 interaction of the adenine moiety in ATP. Additionally, the triphosphate group of ATP established multiple hydrogen bonds with Gly255, Gly257, and Asp258, a pattern also observed with Hits 1–10. Salt bridges with Arg108 further stabilized ATP within the binding site, ensuring proper orientation for catalysis. Conversely, D-fructose binds within a sterically constrained pocket, where it is stabilized by a bidentate interaction between its HO-3′ and HO-4′ groups with Asp15, along with hydrogen bonds involving HO-1′ and Asn45, HO-4′ and HO-5′ with Gly41 and Asn42, and HO-6′ with Glu29B. These interactions play a crucial role in maintaining fructose in an optimal position for phosphorylation. Among the key catalytic residues, Asp258 functions as a conjugate base, extracting the 1-OH proton from D-fructose to facilitate a 1-O-nucleophilic attack on the γ-phosphate of ATP. Meanwhile, Arg108 serves a dual function by stabilizing ATP’s negatively charged phosphate groups and ensuring their correct alignment for efficient phosphate transfer. Hits **1–10** exhibited strong interactions with these catalytic residues, effectively obstructing the active site and inhibiting fructose phosphorylation (Sigrell et al., 1998; Gibbs et al., 2010). By sterically hindering ATP binding and disrupting the alignment of D-fructose, these hits interfered with the enzyme’s function. Notably, the identified hits with their extended structures, interacted with both the ATP and fructose-binding sites, forming hydrogen bonds/water bridges with Gly41, Asn42, and Asn45, similar to the interactions observed with fructose. This suggests a dual-inhibition mechanism. This dual-targeting approach could enhance both potency and specificity, making these hits promising candidates for drug development. By simultaneously impairing substrate recognition and catalytic activity, these inhibitors offer a novel therapeutic strategy for enzyme modulation.

Molecular docking simulation is an important computational tool for identification of ligand’s binding pattern and orientation within a specific macromolecular drug target. However, this tool is not sufficient for ranking multiple ligands based on their experimental affinities. In this regard, a successful structure-based drug design approach should rely on accurate prediction of the energetic basis of ligand-target interactions (El Khoury et al., 2019). To that end, it has been reported that, calculating the ligand free binding affinity to a specific target using molecular mechanics integrated with the generalized Born Surface Area (MM-GBSA) method can significantly improve the accuracy of this prediction (Kaus et al., 2015; El Khoury et al., 2019). Accordingly, the free binding affinity of the top-ranked hits 1-10 towards the KHK-C binding site were computed and the results are provided in Table 2. The best MM-GBSA energy value was represented by the most negative score (Alameen et al., 2023). While the reference ligand (PF-06835919) had an MM-GBSA energy value of −56.71 kcal/mol, the MM-GBSA values of the top-ranked hits ranged from −57.06 to −70.69 kcal/mol, suggesting their relatively high affinity for the KHK-C binding pocket. The dihydroquinazoline derivative, hit 5, exhibited the highest binding affinity, with an MM-GBSA energy value of −70.69 kcal/mol. To gain insights into the contributions of various interaction forces to the total binding energy of each hit, their ΔG binding values were decomposed into individual components. As shown in Table 2, van der Waals energy (MM-GBSA dG Bind vdW), electrostatic energy (MM-GBSA dG Bind Coulomb), non-polar solvation energy (MM-GBSA dG Bind Lipo), and, to a lesser extent, hydrogen bonding (MM-GBSA dG Hbond) were the major contributors to the strong binding of all hits as well as the reference ligand. In contrast, covalent energy (MM-GBSA dG Bind Covalent) and electrostatic solvation energy (MM-GBSA dG Bind Solv GB) made unfavorable contributions. The results of this analysis indicate that the identified hits interacted with the KHK-C binding site through forces similar to those of the reference ligand. Notably, hydrophobic interactions, followed by electrostatic interactions, played a key role in the enhanced binding of the top-ranked hits (1–10) and the reference ligand to the target binding pocket. Interestingly, hits incorporating diethoxy groups attached to the benzene ring—hits 5, 6, and 10—exhibited the highest binding affinities (−70.69, −63.35, and −64.39 kcal/mol, respectively). These hits interacted with two hydrophobic loops (Pro246, Pro247, and Pro248) as well as another loop (Ala224, Trp225, Ala226, and Phe260), further supporting the critical role of hydrophobic interactions in strong binding.

To contextualize our findings, a comparative study of binding free energy was conducted to assess the interaction of the identified hits **1–10** with the target relative to reported inhibitors. The identified hits exhibited promising potential, as their binding energies (Table 1) suggested stronger interactions with the target compared to PF-06835919 (−56.71 kcal/mol). Hits such as hit **1** (−61.13 kcal/mol), hit **3** (−61.04 kcal/mol), and particularly hit **5** (−70.69 kcal/mol) showed more favorable binding energies, indicating a stronger affinity for the target. Hit **5** in particular stood out with the lowest binding energy, suggesting a potentially tighter binding than PF-06835919. Additionally, hit **6** (−63.35 kcal/mol) and hit **9** (−60.52 kcal/mol) also exhibited stronger binding than PF-06835919, implying that these compounds may have superior binding affinity and interaction with the target. The IC_50_ values for the reported inhibitors were 0.01 μM for PF-06835919, 0.02 μM for LY-3522348, and 0.012 μM for BI-9787 (Figure 2). Although the IC_50_ values for the identified hits **1–10** had not been experimentally determined, their stronger binding energies suggest they could demonstrate similar or even enhanced potency compared to the reported inhibitors. Stronger binding often correlates with lower IC_50_ values, and thus, the identified hits might have offered better efficacy in inhibiting the target. Furthermore, LY-3522348 (−45.15 kcal/mol) and BI-9787 (−28.98 kcal/mol) exhibited weaker binding affinities than the identified hits, further supporting the potential of these new compounds as more effective inhibitors. Overall, these results suggest that the identified hits represented a promising new class of inhibitors with potentially higher potency and stronger target affinity than the current reported inhibitors.

In the process of drug discovery and development, an undesirable set of Absorption, Distribution, Metabolism, Excretion, and Toxicity (ADMET) properties is the primary reason for a drug candidate’s failure in the clinical phase, rather than its biodynamic activity (Wu et al., 2020). Therefore, evaluating these properties as early as possible can reduce the drug attrition rate and minimize both time and cost (Cook et al., 2014; Yamashita and Hashida, 2004). In this study, we used the QikProp module embedded in the Schrödinger molecular modeling software to evaluate the pharmacokinetics and drug-like properties of the top-ranked hits **1–10**. This module estimates the ADMET properties of a drug candidate by generating physically relevant descriptors (Ntie-Kang et al., 2013; Schrödinger Press. QikProp 3.4 user manual. New York). As shown in Supplementary Table S1, the overall ADME-compliance scores (#stars) of the top-ranked hits were within the acceptable limit (#stars = 0), suggesting that their property descriptor values fell within the permissible range of corresponding values for approved drugs. One critical aspect of modern drug development is evaluating a drug candidate’s bioavailability in the early stages of discovery, as this directly influences its pharmacological efficacy (Stielow et al., 2024). Efficient drug absorption depends on several parameters, including lipid/water solubility, permeability, interactions with specific transporters in the gut wall, and the metabolic processes that occur before the drug is absorbed into systemic circulation (Caldwell et al., 1995). The calculated descriptors employed for the evaluation of oral bioavailability are the predicted aqueous solubility (QPlogS), the predicted apparent Caco-2 cell permeability in nm/sec (QPPCaco), the predicted human oral absorption (% scale) and adherence to ‘Rule of Three’ (Jorgensen’s rule). Moreover, the rigidity of a molecule defined by the number of rotatable bonds (#rotor) can also affect its oral absorption (Veber et al., 2002). As depicted in Supplementary Figure S1, the top-ranked hits displayed calculated values for these parameters that fell within the specified limits. Therefore, the identified hits are expected to demonstrate high oral bioavailability (>80%), except for hit **4,** which is expected to show average oral bioavailability (<70%). However, this can easily be improved using the prodrug approach by modifying the–NH_2_ group attached to the C2 position of the thiazole ring (Jornada et al., 2015). Our identified hits exhibited excellent predicted oral bioavailability, ranging from 80% to 100%. This surpasses the experimentally determined bioavailability of BI-9787 (68% in rats) and is comparable to or even better than the clinical candidate LY-3522348 (87%) and PF-06835919 (95% in rats). These findings underscore the strong potential of our hits as promising orally available drug candidates (Futatsugi et al., 2020; Durham et al., 2023; Heine et al., 2024). We then investigated the potential of these hits to penetrate the blood-brain barrier (BBB) using computational metrics, including the brain/blood partition coefficient (QPlogBB) and the predicted apparent MDCK cell permeability (QPPMDCK) as additional parameters. Hits **1, 3, 6**, and **7** exhibited moderate BBB penetration (QPlogBB values of −1 to 0), while the remaining hits were predicted to show a very low rate of BBB penetration (QPlogBB values <−1). It was also observed that the apparent MDCK cell permeability of hits **1, 3, 6,** and **7** (Supplementary Figure S1) was greater than 500, suggesting their potential to cross the BBB. Since the site of action of the identified hits is the liver (Maryanoff et al., 2011), achieving therapeutic blood levels in the CNS is not essential in this case. PF-06835919 and LY-3522348, both clinical candidates, were evaluated for their potential to cross the BBB, with QPlogBB values of −0.378 and 0.029, respectively, indicating moderate to low BBB penetration and a low risk of CNS-related off-target effects. In comparison, hits **1–10** exhibited even lower BBB permeability, with values ranging from −0.52 to −1.81, significantly reducing the likelihood of CNS involvement. This suggests that our hits are more likely to be specific to their hepatic target, offering a potentially lower risk of neurological side effects. Thus, our compounds are expected to demonstrate a more focused pharmacological profile, enhancing their therapeutic potential while minimizing unwanted CNS effects. However, further optimization may be required to fine-tune their properties and prevent any potential off-target effects, ensuring a more selective and safer therapeutic profile. To get a meaningful comparison with existing inhibitors, we conducted a comparison of the permeability profiles of our hits. Our hits exhibited superior membrane permeability, with predicted MDCK cell permeability values ranging from 113 nm/s to 2,242 nm/s, indicating enhanced intestinal absorption. In contrast, LY-3522348 showed a modest transport percentage of 4.4% in MDCK cells. BI-9787, with a PAMPA (Parallel Artificial Membrane Permeability Assay) of 4.0 × 10^−6^ cm/s at pH 7.4, demonstrated low passive permeability. This suggests that BI-9787 may require transporter-mediated uptake for effective absorption. The higher permeability of our hits suggests they could offer improved oral bioavailability and systemic uptake, highlighting their potential as promising therapeutic candidates. The extent of drug binding to plasma proteins in the bloodstream has a significant impact on its pharmacological effects. The bioactive fraction is the unbound fraction, which is capable of crossing biological membranes and is therefore subject to biotransformation and excretion (Smith et al., 2010). The extent to which hits 1–10 bind to plasma proteins was estimated using the QPlogKhsa parameter (predicted binding to human serum albumin). High levels of human serum albumin in the blood impact the transportation and distribution of drugs and endogenous substances. From the data provided in Supplementary Table S1, it was noted that our identified hits **1–10** are expected to exhibit weak binding to human serum albumin, as indicated by their low QPlogKhsa values. In contrast, BI-9787 has more than 99% plasma protein binding, and LY-3522348 is 85% bound. The lower plasma protein binding of our hits suggests a higher free drug fraction, which could enhance their effective therapeutic potential by improving distribution and target engagement. This reduced protein binding may facilitate greater systemic exposure, leading to improved bioavailability and more efficient drug delivery to the target site. Additionally, a higher free drug concentration in plasma can contribute to a more rapid onset of action, potentially allowing for lower effective doses and reducing the risk of off-target interactions associated with high plasma protein binding. These findings highlight the potential pharmacokinetic advantages of our identified hits over existing inhibitors, warranting further investigation into their *in vivo* behavior and therapeutic efficacy (Durham et al., 2023; Heine et al., 2024). The estimated number of potential metabolic reactions for each hit was also evaluated, as this will affect the rate at which a drug candidate reaches its site of action immediately after entering the blood circulation. As depicted in Supplementary Table S1, the number of likely metabolic reactions for hits **1–10** (#metab) were within the specified limit (1-8), demonstrating their potential to reach their site of action in their intact forms. The predicted number of metabolic reactions for hits **1–10** varies between 4 and 7, indicating that these compounds are expected to undergo a moderate degree of metabolism in the liver. This suggests a relatively stable metabolic profile, which is generally favorable for maintaining pharmacokinetic properties such as bioavailability. However, when compared to LY-3522348, our hits demonstrated slightly lower metabolic stability. LY-3522348, as reported by Elsaman et al., is predicted to undergo only 1 metabolic reaction, which is notably lower than the metabolic activity observed for our compounds. Moreover, LY-3522348 has been reported to show 0 metabolic reactions following a 30-min incubation in liver microsomes, further confirming its high metabolic stability (Durham et al., 2023; Elsaman and Mohamed, 2025). Thus, our hits may require further optimization to improve their metabolic stability and overall pharmacokinetic profiles. Next, we computed the parameter related to the likelihood of hits **1–10** to be cardiotoxic. It has been reported that blocking the hERG (human Ether-à-go-go Related Gene) K^+^ channel by the drugs delays the cardiac repolarization step resulting in fatal type of arrhythmia called *torsade de pointes* (long QT syndrome) (Napolitano et al., 1994). In this study, the estimated IC_50_ values for blockade of this channel by hits 1-10 (QPlogHERG) are given in Supplementary Table S1. Accordingly, we evaluated the hERG inhibition potential of our hits in comparison to existing inhibitors, PF-06835919 and LY-3522348. PF-06835919 shows a hERG IC_50_ > 30, considered safe for cardiac toxicity, while our hits **1, 2, 4–6** exhibit predicted IC_50_ values above −5, suggesting a lower likelihood of hERG channel inhibition. In contrast, hits **3, 7–10** were identified as potentially cardiotoxic, as their IC_50_ values for blocking hERG K^+^ channels exceeded the permissible range (>−5) and were excluded from further investigations. The calculated hERG value for LY-3522348 by Elsaman et al. was −4.6, confirming its minimal cardiac toxicity risk, with tissue distribution studies in rats showing no significant cardiotoxicity, supporting its favorable safety profile. Thus, some of our hits may require further optimization to reduce cardiotoxicity risk (Durham et al., 2023; Zhu et al., 2023; Elsaman and Mohamed, 2025).

In order to reduce the risk of cardiotoxicity associated with the excluded hits **3**, **7–10**, scaffold replacement was performed for each compound, considering key factors such as reducing lipophilicity, decreasing planarity, minimizing basicity, and enhancing hydrogen bonding capacity. These modifications were aimed at lowering the compounds’ hERG channel binding affinity while maintaining their pharmacological potential and ensuring favorable drug-like properties. A total of ten replacements were performed for each excluded hit, using various functional modifications carefully chosen to optimize the balance between efficacy and safety. Among these, some replacements successfully reduced cardiotoxicity while others resulted in a decrease in binding affinity. Notably, only three analogs (Figure 11) showed a significant reduction in cardiotoxicity. For hit **8**, the replacement of the pyrazine ring with the bicyclo [2.2.1]heptane structure successfully reduced the cardiotoxicity to the specified limit and resulted in hit **8a**, which showed a docking score of −8.01 kcal/mol. The first analog for hit **10** (hit **10a**) involved replacing piperazine with 1,2,3-triazole, which resulted in a docking score of −7.8 kcal/mol, still higher than the co-crystal ligand, suggesting a strong binding affinity to the target despite the modification. This change was made to reduce the basicity of the molecule and disrupt its planarity, thereby minimizing interactions with the hERG channel. The second analog for hit **10** (hit **10b**) featured the replacement of piperazine with 2,5-diketopiperazine, which had a docking score of −8.4 kcal/mol. This modification reduced basicity and increased polarity, which was expected to decrease lipophilicity and improve the molecule’s solubility profile. This replacement led to a noticeable reduction in cardiotoxicity, making it a promising candidate for further optimization. These scaffold modifications have shown the potential to significantly mitigate cardiotoxicity while retaining favorable docking scores, making them suitable candidates for further *in vitro* and *in vivo* evaluation.

KHK-C inhibitors have the potential to induce mechanism-linked toxicological consequences by disrupting fructose metabolism, an important factor that should be carefully considered (Koene et al., 2025). KHK-C inhibitors disrupt fructose metabolism, potentially causing adverse effects such as fructosuria, osmotic diuresis, and glycogen accumulation (Park et al., 2024). Elevated urinary fructose may promote bacterial growth, increasing the risk of recurrent *Escherichia coli* infections. Additionally, excess fructose in the gastrointestinal tract can contribute to dysbiosis, bacterial overgrowth, and metabolic disturbances (Pinheiro et al., 2020; Jung et al., 2022). Increased circulating fructose levels may lead to oxidative stress, endothelial dysfunction, and systemic imbalances (Herman and Birnbaum, 2021). KHK-C inhibition may also divert fructose metabolism towards the sorbitol and xylulose pathways, potentially leading to sorbitol accumulation and complications in diabetic patients (Andres-Hernando et al., 2017). In general, the outcome of this analysis indicates that hits **1,2** and **4-6** displayed acceptable drug-likeness, pharmacokinetics features, and safety profiles, thereby denoting their potential as KHK-C inhibitors for future treatment of fructose metabolic diseases.

Based on the predicted ADMET properties discussed in the previous section, hits **1**, **2** and **4-6** which demonstrated positive drug-likeness and pharmacokinetics properties, were chosen for 100 ns MD simulation. MD simulation is a powerful computational tool frequently utilized to validate the stability of protein-ligand complexes and the nature of the ligand’s key binding interactions in a dynamic condition (AlRawashdeh and Barakat, 2024). In this context, the stability of the best docked conformations of the investigated hits and the reference ligand (PF-06835919) were monitored in aqueous system using Desmond package (Schrödinger Release, 2023: Desmond Molecular Dynamics System). Root Mean Square Deviation (RMSD) values of the protein C-α atoms relative to the initial structure were used to evaluate the stability of the studied complexes (De Vivo et al., 2016). As seen in Table 3 and Figure 11, the C-α of the reference ligand complex backbone exhibited an average RMSD of 2.41 ± 0.43 Å over time. Unstable fluctuation behavior was observed throughout the simulated time, indicating that the complex did not achieve a steady state profile. On the other hand, the simulated system of hits **1, 2** displayed average RMSD values of 2.47 ± 0.31 Å and 2.34 ± 0.27 Å, respectively. These systems were unstable during the initial frames up to 15 ns, then the systems converged and they attained the equilibrium state, and protein fluctuations were maintained in the range of 1.89–2.99 Å throughout the simulation run. For the hit **4** complex, the average C-α RMSD value was 2.24 ± 0.34 Å as a function of time and the protein showed drifts up to 40 ns, and remained stable during the remainder of the simulation time, maintained in the range of 1.63–3.04 Å. Conversely, hit **5** reached equilibrium quickly (average RMSD = 2.19 ± 0.41 Å) and remained stable between 1.25 Å and 2.56 Å until 65 ns, after which relatively high deviations were observed for the rest of the simulation. The average C-α RMSD value of the hit **6** complex was 2.36 ± 0.38 Å over time. Minor perturbations were noted for this complex during the 100 ns simulation, reaching a steady state around 23 ns? The deviation ranged from 1.644 Å to 3.26 Å. Overall, our findings indicated that the structure of the KHK-C did not experience a significant shift and remained stable following complexation with hits **1, 2,** and **4–6**. The C-α RMSD profiles of these complexes were better that of the reference ligand, suggesting their potential as KHK-C inhibitors. Next, we performed Root Mean Squire Fluctuation (RMSF) analysis for each ligand-bound KHK-C to assess the mobility of the target’s individual residues during the trajectory run. RMSF defines the local variability in the protein C*α*-atoms, evaluating its flexibility and stiffness from trajectories recorded during a 100 ns MD simulations timescale (Khan et al., 2022). A higher RMSF values suggests significant mobility, whereas a lower RMSF values indicates a stable structure with limited fluctuations (Stofberg et al., 2024). The RMSF for the simulated systems of the docked complexes of the top-ranked hits **1,2**, and **4-6**, as well as the reference ligand for comparison are graphically depicted in Figure 12. All of the simulated systems fluctuate strongly mainly in two regions (>3 Å), (i) amino acid residues Lys22-Arg31, and (ii) amino acid residues Asn102-Asn107, fortunately, these residues are neither critical for enzyme inhibition nor directly involved in protein-ligand interactions (Maryanoff et al., 2011; Huard et al., 2017; Futatsugi et al., 2020; Zhu et al., 2023). On the other side, the crucial residues exist in the ATP binding site, (Phe245, Pro246, Pro247, Thr253, Gly255, Gly257, Phe260 and Cys282), experienced limited conformational mobility (RMSD <1.5 Å) in the studies complexes across the simulation timescale. Interestingly, hits **2**, **5**, and **6** enhanced the stability of Arg108, a residue previously reported as critical for maintaining potency, compared to the reference ligand (Huard et al., 2017; Zhu et al., 2023). In general, the RMSF profiles of the investigated hits showed a degree of stability for those residues comparable to or higher than that of the reference ligand, pointing to their potentially strong KHK-C inhibitory properties. Consequently, we conducted protein-ligand interaction analysis to gain insights into the types and strengths of intermolecular interaction forces during the simulation process. As shown in Supplementary Figures S4–S9, different types of non-bonding interaction forces were involved in the ligand–protein complex overall stability. These forces comprised H-Bonds, hydrophobic contacts, electrostatic interactions, and Water Bridges. In the case of the reference ligand (Supplementary Figure S4), it retained the same conformation shown in molecular docking study, indicating its stability in the enzymes binding site. Moreover, it exhibited stable Water Bridge interactions with the critical residues Phe245 and Cys282, which persisted for >90% of the simulation time. In addition, the carboxylate anion formed multiple interactions with key residues including Gly255, Gly257 and ASH258, which were stable for <30% of the simulation run. Furthermore, new H-Bonds were seen between the carboxylate anion and Arg108 as well as Thr253, which were stable for <15% of the simulation time. Finally, moderate hydrophobic interactions with Ala226 and Phe260 were also noticed during the simulation timescale. These results are consistent with the previous report describing the critical interactions during MD simulation for potent KHK-C inhibitors (Zhu et al., 2023). In case of hit **1** (Supplementary Figure S5), the dimethoxy groups involved in multiple Water Bridges formation with Cys282 and Phe245, while the amide carbonyl group existed in the linker created similar interactions with Thr253, Gly255, Ala256 and Gly257. Importantly, the Water Bridges formed with the critical residue Phe245 was not present in the docked conformer, pointing to the fact that this hit underwent minor conformational changes during MD simulations favoring these interactions. Generally, the hit **1** contacts with the KHK-C binding pocket were less strong (interaction fraction <1) than the reference ligand, suggesting its potential low KHK-C inhibitory activity. Regarding hit **2**, as shown in Supplementary Figure S6, it retained all of the non-bonding interactions observed in molecular docking study (Figure 8). Additionally, three new Water Bridges were detected with the crucial residues Phe245 and Cys282, established by the oxygen atoms of the 3, 4 dimethoxy groups attached to the phenyl group. The Water Bridges formed by the 3-methoxy group persisted for >95% of the simulation time, while those made by the 4-methoxy group covered >65% of the simulation timeframe. The interaction fractions of these bonds for hit **2** were >1.5, indicating stronger interactions profile as compared to the reference ligand and the previously reported KHK-C inhibitor (<1) (Zhu et al., 2023). In addition to these bonds, hit **2** formed H-Bonds and Water Bridges interactions with the key residues Glu227, Gly255, Gly257 and ASH258 at the solvent exposed opening of the enzyme binding pocket. These bonds were similar in their strength to those of the reference ligand. Unlike the reference ligand, a new moderately stable H-Bond was observed with the key residue Thr253, which persisted for 40% of the simulation run, higher than that for the previously reported potent KHK-C inhibitor (Zhu et al., 2023). Furthermore, the methyl group of the 5-methoxy group linked to the phenyl ring filled the sub pocket made by Phe260 which was filled by the methyl group of the azetidine ring in case of reference ligand. This filling established hydrophobic interactions of similar strength in both complexes contributing to their overall stability. On the other hand, the interaction of hit **2** with Arg108, a residue that considered critical for optimum potency (Futatsugi et al., 2020), was minimal as compared to that of the reference ligand. This could be justified by the fact that the reference ligand incorporated an ionized carboxylate which seemed efficient in forming interactions with the guanidinium ion of the Arg108**.** Consequently, we designed a hit **2** analog, shown in Figure 13, by introducing free carboxylate group at the terminal side chain to create a new ionic bond with Arg108. Glide XP docking of the designed analog into the KHK-C catalytic site resulted in an improved docking score (−9.23 kcal/mol) and the formation of an ionic bond (3.29 Å distance) alongside two H-Bonds (2.55 Å and 2.65 Å) between the terminal carboxylate group and the key residue Arg108. This modification could enhance the binding affinity of hit **2** and consequently improve its overall efficacy. Further MD simulation studies are needed to confirm the stability of the interaction map of the designed analog. Regarding hits **4–6** (Supplementary Figures 7–9), they demonstrated relatively weak binding interactions during MD simulations with the key residues Phe245 and Cys282 (hits **5** and **6**)**,** or Gly255 and Gly257 (hit **4**), compared to the reference ligand, suggesting their lower potential for KHK-C inhibition. Generally speaking, hit **2** interacted with the most critical residues in the enzyme binding pocket and created intermolecular bonds that were stronger than those made by reference ligand. Moreover, hit **2** did not engage in binding interactions with residues conserved in KHK-A (Asp15, Asn42, Asn45, Arg141, and Lys174) (Ferreira et al., 2024), indicating its potential selectivity for targeting KHK-C. These facts indicate that hit **2** could be a strong and selective KHK-C inhibitor. Following this step, we turned to analyze the number of H-Bonds, Water Bridges and hydrophobic interactions that stabilized the binding of the hit **2** to the KHK-C catalytic site throughout the simulation course, compared to the reference ligand. As indicated in Table 4 and Figure 15A, hit **2** formed similar number of H-Bonds with the enzyme’s binding site residues like the reference ligand averaging 1.15 and 1.52, respectively. However, the number of Water Bridges established by hit **2** (Table 4; Figure 15B) were nearly twice those formed by the reference ligand averaging 5.5 and 2.9, respectively. This could justify the greater stability and higher affinity of hit **2** compared to the co-crystalized ligand, as H-bonds are increasingly considered as facilitators of protein-ligand complexation (Chen et al., 2016). Likewise, hit **2** exhibited high number of hydrophobic contacts (Table 4; Figure 15C) as compared to the reference ligand averaging 1.47 and 0.64, respectively. While Phe260 was the most frequently involved in hydrophobic interaction with the reference ligand, multiple residues existed in the active site were involved in this type of interactions with hit **2**. Collectively, the results suggested that hit **2** demonstrated more stable interactions compared to the reference ligand, highlighting its potential as a potent KHK-C inhibitor worthy of experimental validation.

The RMSF analysis of the co-crystal ligand and hit **2** (Figure 16) was performed to assess their movement and stability within the binding pocket of KHK-C, providing insights into their flexibility, conformational changes, and potential interactions with key residues throughout the simulation. The analysis of key atom fluctuations in the RMSF data of the co-crystallized ligand focused on specific atoms and their interactions. The carboxylate oxygens showed high fluctuations (∼2.73 Å) (Figure 16A), despite forming strong hydrogen bonds with Gly255 and Gly257, ionic bonds with Arg108, and water-bridge interactions with Asn58. This suggests dynamic behavior, potentially resulting from the increased flexibility of the carboxylate group as it orients toward the solvent-exposed region, allowing for greater mobility. In addition, the CF_3_ group fluorines exhibited increased fluctuations (∼1.2–2.3 Å), despite typically being restricted by hydrophobic interactions with the Proline Loop (Pro246–Pro248). The fluctuations likely indicate conformational shifts in the proline loop that affect ligand positioning, with the elevated RMSF of the CF_3_ group suggesting potential loop adaptation. To this end, hydrogen bond occupancy analysis revealed that interactions with Gly255, Gly257, and Arg108 were intermittent (Supplementary Figure S4), with occupancy below 20%. This indicated that these hydrogen bonds were transient and frequently disrupted, likely due to high fluctuations of the carboxylate group, which affected its ability to maintain stable interactions. Additionally, the water-bridge interaction with Asn58 exhibited a short lifetime, suggesting weak and dynamic water-mediated stabilization rather than a persistent interaction. In contrast, the co-crystallized ligand maintained stable water-bridge interactions with the critical residues Cys282 and Phe245. These interactions, mediated by the nitrogen (Atom 3) (Figure 15A) of the pyrimidine ring, were present for most of the simulation time. The persistent nature of these water bridges suggested a key stabilizing role in ligand binding, likely compensating for the weaker direct hydrogen bonds with other residues. This stability contributed to maintaining the co-crystallized ligand’s position within the active site of KHK-C, reinforcing its overall binding affinity. In hit **2**, the interactions of key atoms with the protein correlated with their RMSF values, highlighting the dynamic behavior within the binding pocket. Atoms 2 and 3 (oxygens) (Figure 16B) showed flexibility below 1 Å and participated in multiple water-bridge interactions with Cys282 and Phe245. These interactions showed limited fluctuations, suggesting dynamic but stabilizing water-mediated hydrogen bonds that helped anchor the ligand. The enhanced stability of O24 and O25, as indicated by the hydrogen bond occupancy analysis, led to the formation of multiple stable water-bridge interactions with the critical residues Cys282 and Phe245, with fluctuations below 1 Å and persisting for more than 95% of the simulation run (Supplementary Figure S6). This resulted in stronger interactions compared to the co-crystallized ligand and contributed to a more stable binding mode, improving the overall ligand stability within the binding pocket. Methyl groups 24, 25, and 26 interacted with the hydrophobic pocket formed by the proline loop (Pro246–Pro248). The RMSF values of these atoms indicated limited flexibility (below 1.2 Å), and methyl group 26 filled the subpocket formed by Phe260, allowing for ligand accommodation and stabilizing interactions. This was reflected in enhanced hydrophobic interactions of Hit **2** compared to the reference ligand, further contributing to its binding stability. Atom 5 (oxygen) formed a hydrogen bond with Thr253, showing a high RMSF of 2.6 Å, indicating that the interaction is intermittent (below 50%) (Supplementary Figure S6). However, it remains better than the interaction observed in the co-crystallized ligand, suggesting a more dynamic yet still effective binding. Atom 8 (nitrogen), interacting with Glu227, showed a low RMSF of 1.3 Å, indicating a stable interaction crucial for maintaining ligand positioning. The terminal methoxy group of the side chain (atoms 1 and 23) showed the highest flexibility, with fluctuations exceeding 4 Å, as they oriented towards the solvent-exposed area, allowing for greater mobility and fluctuating interactions with the surrounding environment. Overall, the interacting groups of hit **2** with the enzyme KHK-C displayed minimal flexibility, particularly the water-bridge and hydrophobic interactions, which contributed to the stable binding of the ligand within the binding site. The limited fluctuations observed in key atoms, along with the persistent water-mediated interactions, helped anchor the ligand more firmly in the pocket, resulting in a stable binding mode. This stability is crucial for maintaining optimal interactions with critical residues and enhancing the overall affinity of the ligand for the enzyme, thereby supporting its potential as a promising inhibitor. In order to further investigate the stability and movement of Hit 2 within the KHK-C binding site, we conducted a radius of gyration (rGyr) analysis over a 100 ns simulation to assess its structural compactness and flexibility, including the reference ligand for comparison. The radius of gyration (rGyr) analysis over the 100 ns simulation, as shown in Figure 17, provides critical insights into the structural stability of ligands within the KHK-C binding site. The reference ligand maintained a compact conformation with an average rGyr of 4 Å, indicating stable binding within the active site with minimal fluctuations. In contrast, Hit 2 exhibited a higher rGyr, fluctuating between 5.5 and 6 Å, suggesting a more extended conformation. These fluctuations, observed in Figure 16, implied greater flexibility within the KHK-C binding pocket, which may have allowed dynamic interactions with key catalytic residues. The reference ligand’s lower rGyr suggested strong stabilization within the enzyme’s active site, likely forming rigid and well-defined interactions. Hit 2’s flexibility could have facilitated the exploration of additional binding modes, potentially enhancing interactions with crucial residues. Both ligands demonstrated overall structural stability, as no significant deviations or drastic conformational changes were observed throughout the simulation. The variations in rGyr, illustrated in Figure 16, suggest potential differences in ligand accommodation, which might have influenced binding affinity and stability. The ability of **h**it 2 to adopt multiple conformations within KHK-C could be relevant for optimizing its binding efficiency. This flexibility is facilitated by the presence of multiple rotatable bonds, which enhance its structural flexibility, whereas the reference ligand contains a rigid multicyclic structure. This analysis confirms that **h**it 2 maintained its integrity within the KHK-C catalytic site, supporting further investigation into its binding mechanisms and potential inhibitory effects.

To assess the potential of hit **2** in inhibiting the KHK-A isoform, we first compared its performance with PF-06835919 in KHK-C. In KHK-C, hit **2** demonstrated a significantly higher binding affinity than PF-06835919, with a docking score of **‒**9.07 and a binding affinity of **‒**59.61 kcal/mol, compared to PF-06835919s docking score of **‒**7.76 and binding affinity of **‒**56.71 kcal/mol. Furthermore, hit **2** exhibited dynamically stable behavior, forming a more stable complex and establishing stronger interactions with critical residues, both in type and intensity, compared to PF-06835919. Docking studies for KHK-A were carried out using the crystal structure (PDB ID: **8OME**), following the same docking protocols used for KHK-C. The results revealed that Hit **2** displayed a considerably stronger binding affinity towards KHK-A, with a binding free energy of **‒**56.8 kcal/mol and a docking score of −6.79, surpassing PF-06835919, which showed a binding affinity of 3.49 kcal/mol and a docking score of **‒**4.36. Notably, PF-06835919 exhibited approximately six-fold higher inhibitory properties for KHK-C compared to KHK-A, and the predicted binding affinities in the present study align with this trend (Gutierrez et al., 2021). KHK-A selective inhibition allows more fructose to be delivered to the liver, leading to increased KHK-C activity and greater fructose metabolism. However, dual inhibition of both KHK-A and KHK-C may provide superior metabolic benefits. Studies have shown that KHK-A/C knockout (KO) mice are protected from the metabolic effects of fructose, highlighting the potential advantages of targeting both isoforms (Ishimoto et al., 2012). Overall, these findings underscore hit **2**’s superior binding affinity, stability, and ability to interact with key residues in both KHK-C and KHK-A, positioning it as a highly promising candidate for further development as an inhibitor for both isoforms.

### 4.1 Study limitations and future perspectives

Despite the valuable insights gained from this study, several limitations must be acknowledged. First, computational techniques such as molecular docking and free energy calculations, while powerful, have inherent constraints. These methods may not fully account for the dynamic nature of protein-ligand interactions, including protein flexibility, solvent effects, and entropic contributions. Additionally, the MD simulations were limited to 100 ns, which, although sufficient for capturing short-to mid-term stability, may not fully represent long-term conformational shifts and rare binding events. More extended simulations could provide a deeper understanding of the ligand’s stability and binding mechanisms. Second, the findings are based entirely on computational predictions without experimental validation. Enzyme inhibition assays and cell based assays are necessary to confirm the inhibitory activity of the identified compounds against KHK-C, while *in vivo* studies are crucial for assessing their pharmacokinetic properties, metabolic stability, and overall therapeutic potential. Without these experimental confirmations, the transition from computational discovery to drug development remains uncertain. Third, some of the identified compounds exhibited physicochemical properties that suggest potential penetration of the BBB. This raises concerns about unintended neurological effects, which could impact their safety profile. Lastly, the potential cardiotoxicity of some identified hits remains a concern. Certain compounds may interfere with the hERG channel, increasing the risk of QT interval prolongation and associated cardiac arrhythmias. To overcome these limitations, future studies should include experimental validation, such as enzyme inhibition assays, *in vivo* ADMET profiling, and toxicity assessments. Additionally, incorporating extended molecular dynamics simulations will provide a more thorough understanding of the long-term dynamics and binding stability of the lead compounds. These comprehensive approaches will help refine the identified hits and move the most promising candidates forward for preclinical evaluation and potential therapeutic use.

## 5 Conclusion

This study aims to identify novel and potent KHK-C inhibitors for treating fructose-related metabolic disorders, including obesity, diabetes, NAFLD, and NASH. Despite the high prevalence of these conditions, no clinically approved drugs specifically target KHK-C, although PF-06835919 and LY-3522348 are currently in phase II clinical trials. Virtual screening of the NCI library (460,000 small molecules) using various computational tools identified ten potential candidates with docking scores and binding energies surpassing those of PF-06835919 and LY-3522348. Further filtration through ADMET profiling and molecular dynamics simulations highlighted compound 2, (E)-N-(3-methoxypropyl)-3-oxo-3-(2-(3,4,5-trimethoxybenzylidene)hydrazineyl)propanamide, as the most promising candidate, warranting further experimental validation.

## Data Availability

The original contributions presented in the study are included in the article/Supplementary Material, further inquiries can be directed to the corresponding authors.
