# Supplementary material for: Pharmacophore-based virtual screening and in silico investigations of small molecule library for discovery of human hepatic ketohexokinase inhibitors for the treatment of fructose metabolic disorders
Source: Front Pharmacol. 2025 Apr 7;16:1531512. doi: 10.3389/fphar.2025.1531512 (PMC12009819; doi:10.3389/fphar.2025.1531512)
Supplement: Supplementary file 1 [file DataSheet1.docx]

**Pharmacophore-Based Virtual Screening and In Silico Investigations of Small Molecule Library for Discovery of Human Hepatic Ketohexokinase Inhibitors for the Treatment of Fructose Metabolic Disorders**

**Tilal Elsaman*^1^, Magdi Awadalla Mohamed*^1^, Abozer Y. Elderdery^2^, Abdullah Alsrhani^2^, Badr Alzahrani^2^, Heba Bassiony Ghanem^2^, Jeremy Mills^3^, Musaed Rayzah^4^, Nasser A. N. Alzerwi^4^, Afnan Al-sultan^5^, Bandar Idrees^6^, Fares Rayzah^7^**

^1^*Department of Pharmaceutical Chemistry, College of Pharmacy, Jouf University, Sakaka, Saudi Arabia*

*^2^Department of Clinical Laboratory Sciences, College of Applied Medical Sciences, Jouf University, Sakaka, Saudi Arabia*

*^3^ School of Medicine, Pharmacy and Biomedical Sciences, University of Portsmouth, Portsmouth, United Kingdom*

*^4^Department of Surgery, College of Medicine, Majmaah University, Al Majma’ah, Saudi Arabia*

*^5^Department of surgery, King Saud Medical City, Riyadh, Saudi Arabia*

*^6^Department of Surgery, Prince Sultan Military Medical City, As Sulimaniyah, Saudi Arabia*

*^7^* *Department of Surgery Aseer Central Hospital, Abha, Saudi Arabia*

*Authors to whom correspondence should be addressed; [telbashir@ju.edu.sa](mailto:telbashir@ju.edu.sa) and [maelhussein@ju.edu.sa](mailto:maelhussein@ju.edu.sa)

| **Supplementary Table S1: ADMET and drug-like properties of the top 10 hits determined by Qikprop module of Schrödinger** | | | | | | | | | | | | |
| --- | --- | --- | --- | --- | --- | --- | --- | --- | --- | --- | --- | --- |
| **Descriptor** | **Description** | **Permissible range** | **1** | **2** | **3** | **4** | **5** | **6** | **7** | **8** | **9** | **10** |
| #stars | Number of property or descriptor values that fall outside the 95% range of similar values for known drugs. | (0–5) | 0 | 0 | 1 | 0 | 0 | 0 | 0 | 0 | 0 | 0 |
| #amide | Number of non-conjugated amide groups | (0–1) | 1 | 1 | 0 | 1 | 1 | 1 | 0 | 0 | 0 | 0 |
| #rotor | Number of non-trivial, non-hindered rotatable bonds | (0–15) | 8 | 12 | 10 | 8 | 12 | 11 | 5 | 7 | 7 | 9 |
| #rtvFG | Number of reactive functional groups | (0–2) | 0 | 0 | 0 | 0 | 0 | 0 | 0 | 0 | 2 | 2 |
| mol_MW | Molecular weight of the molecule. | (130–725) | 396.44 | 367.40 | 402.50 | 337.393 | 485.53 | 439.55 | 427.492 | 316.31 | 417.461 | 445.514 |
| SASA | Total solvent accessible surface area | (300–1000) | 681.49 | 731.52 | 781.67 | 629.02 | 810.154 | 819.04 | 712.178 | 607.98 | 736.393 | 784.271 |
| FOSA | Hydrophobic component of the SASA | (0–750) | 375.73 | 548.44 | 530.15 | 308.23 | 543.26 | 545.10 | 221.836 | 284.21 | 479.87 | 544.752 |
| FISA | Hydrophilic component of the SASA | (7–330) | 94.69 | 116.40 | 72.97 | 187.14 | 131.43 | 81.68 | 95.179 | 116.12 | 185.743 | 159.314 |
| PISA | π (carbon and attached hydrogen) component of the SASA | (0–450) | 211.06 | 66.68 | 145.15 | 96.61 | 135.44 | 192.26 | 294.298 | 207.64 | 70.779 | 80.205 |
| WPSA | Weakly polar component of the SASA | (0–175) | 0 | 0 | 33.39 | 37.02 | 0 | 0 | 101.405 | 0 | 0 | 0 |
| donorHB | Estimated number of hydrogen bonds that would be donated by the solute to water molecules in an aqueous solution. | (0–6) | 1 | 0 | 1 | 3 | 2 | 1 | 1 | 1 | 1 | 1 |
| accptHB | Estimated number of hydrogen bonds that would be accepted by the solute from water molecules in an aqueous solution | (2–20) | 7.75 | 6.95 | 5.25 | 8 | 9 | 6 | 8 | 7.25 | 10 | 10 |
| QPlogPo/w | Predicted octanol/water partition coefficient. | (−2–6.5) | 2.825 | 2.698 | 5.378 | 0.784 | 3.36 | 5.06 | 3.78 | 2.15 | 2.211 | 3.105 |
| QPPCaco | Predicted apparent Caco-2 cell permeability in nm/sec | (<25poor, >500 great) | 812.93 | 494.13 | 2013.23 | 108.11 | 361.01 | 1036.59 | 1239.71 | 784.68 | 171.592 | 305.581 |
| QPlogS | Predicted aqueous solubility | –6.5 – 0.5 | ‒3.725 | ‒3.663 | ‒7.068 | ‒3.026 | ‒4.873 | ‒6.281 | ‒5.883 | ‒3.593 | –4.752 | –5.251 |
| CIQPlogS | Conformation-independent predicted aqueous solubility | –6.5 – 0.5 | ‒4.489 | ‒3.37 | ‒6.01 | ‒2.87 | ‒5.57 | ‒6.00 | ‒5.893 | ‒3.50 | ‒4.142 | –4.701 |
| QPlogBB | Predicted brain/blood partition coefficient. | –3.0 – 1.2 | ‒0.853 | ‒1.481 | ‒0.804 | ‒1.759 | ‒1.602 | ‒1.028 | ‒0.52 | ‒1.034 | ‒1.819 | ‒1.69 |
| QPPMDCK | Predicted apparent MDCK cell permeability in nm/sec. | <25 poor >500 great | 631.24 | 378.16 | 1606.05 | 113.60 | 265.18 | 858.16 | 2242.417 | 380.649 | 73.609 | 137.351 |
| #metab | Number of likely metabolic reactions. | (1 – 8) | 5 | 5 | 7 | 5 | 6 | 6 | 4 | 6 | 4 | 5 |
| QPlogKhsa | Prediction of binding to human serum albumin. | −1.5–1.5 | ‒0.189 | ‒0.45 | ‒0.833 | ‒0.679 | 0.023 | 0.644 | 0.177 | ‒0.295 | ‒0.085 | 0.122 |
| QPlogHERG | Predicted IC_50_ value for blockage of HERG K^+^ channels. | Concern below –5 | ‒4.04 | ‒4.25 | ‒6.13 | ‒3.79 | ‒4.37 | ‒4.86 | ‒6.422 | ‒5.574 | ‒5.19 | ‒5.346 |
| Human Oral Absorption | Predicted qualitative human oral absorption | 1, 2, or 3 for low, medium, or high. | 3 | 3 | 1 | 3 | 3 | 1 | 3 | 3 | 3 | 3 |
| PHOP% | Predicted human oral absorption on 0 to 100% scale. | >80% is high | 95.6 | 91 | 100 | 67.9 | 92.4 | 100 | 100 | 91.3 | 80 | 89.608 |
| PSA | VdW surface area of polar nitrogen and oxygen atoms | 7–200 | 89.04 | 120.46 | 74.83 | 121.98 | 134.39 | 81.05 | 93.675 | 98.25 | 133.007 | 127.474 |
| Rule Of Five | Number of violations of Lipinski’s rule of five | maximum is 4 | 0 | 0 | 1 | 0 | 0 | 1 | 0 | 0 | 0 | 0 |
| Rule Of Three | Number of violations of Jorgensen’s rule of three. | maximum is 3 | 0 | 0 | 2 | 0 | 0 | 1 | 1 | 0 | 0 | 0 |


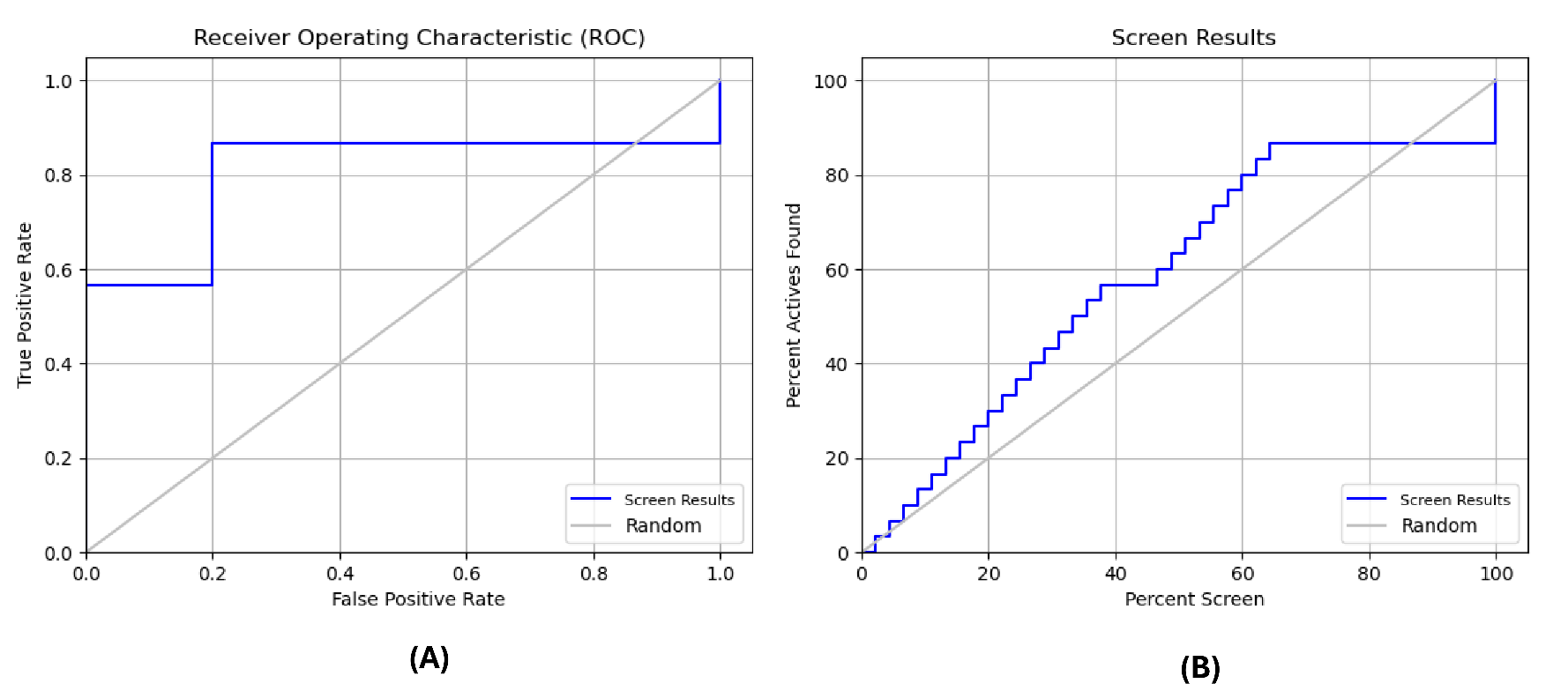


**Supplementary Figure S1. Results of the hypothesis validation, showcasing key performance metrics and screening outcomes. (A) ROC curve for hypothesis validation. (B) Screening results of actives and inactives.**


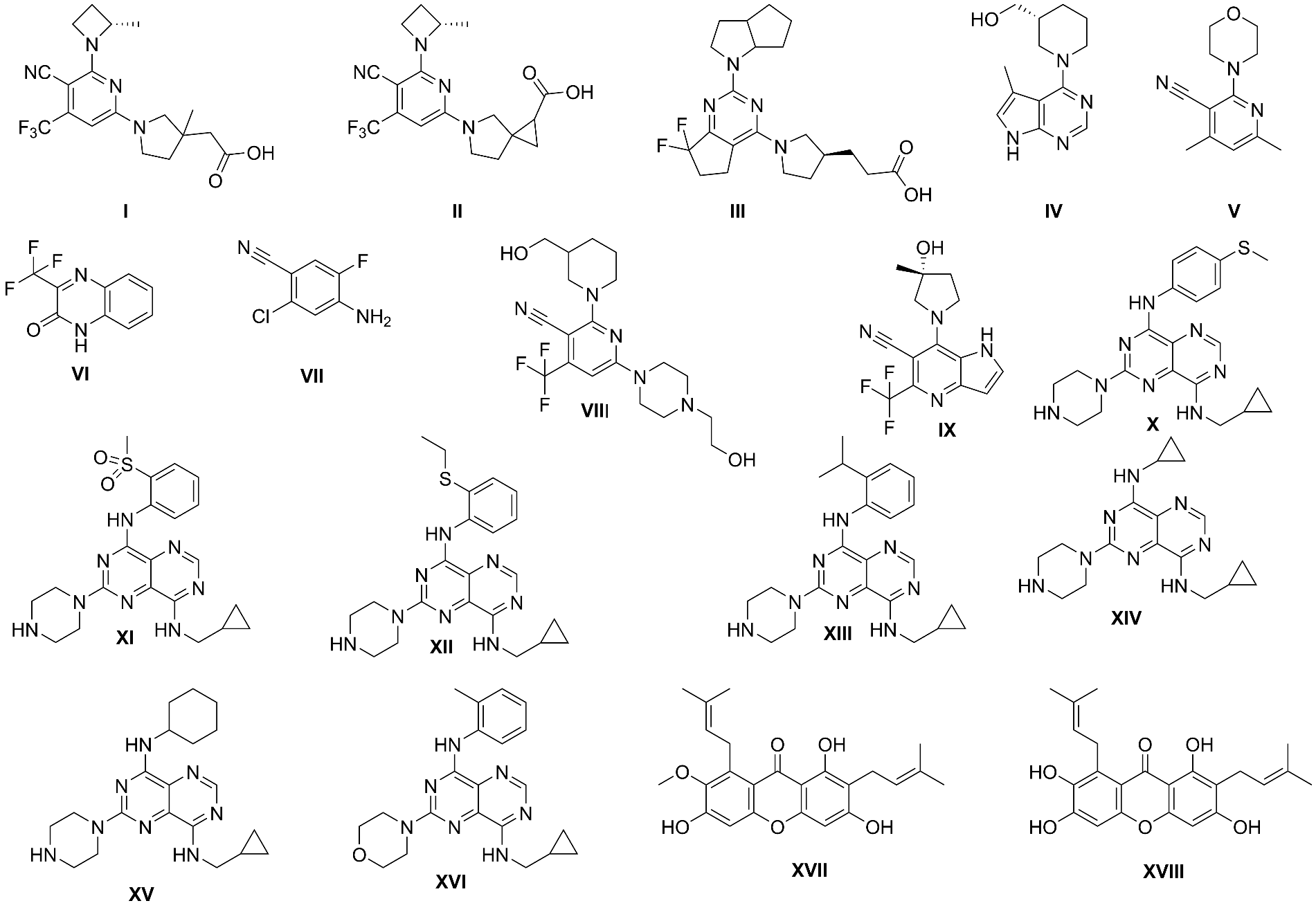


**Supplementary Figure S2. Chemical structures of the KHK-C inactives sourced from the literature with IC₅₀ greater than 1 micromolar.**

| 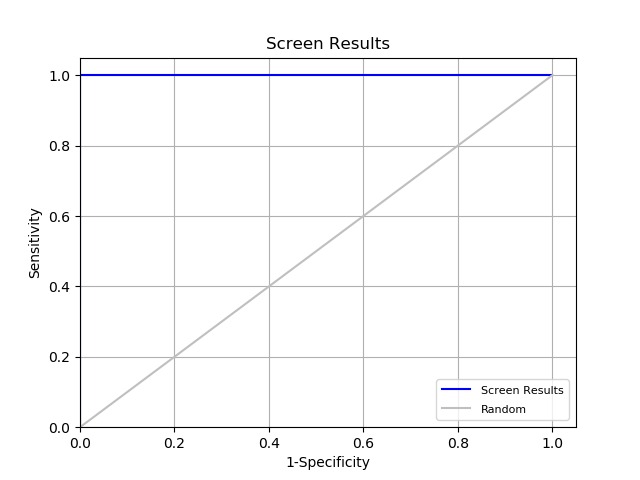 | 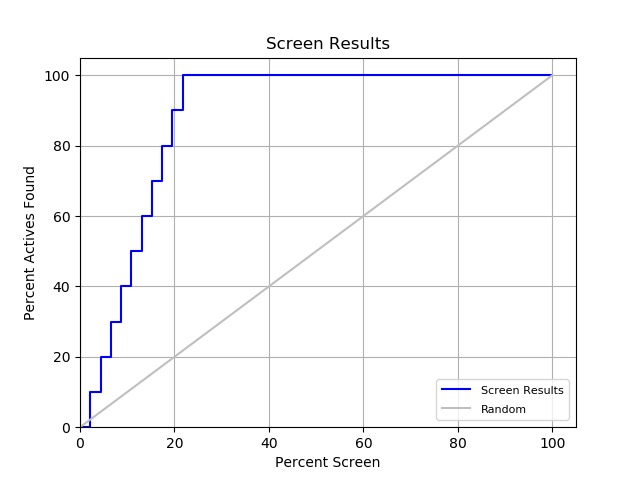 |
| --- | --- |
| **(A)** | **(B)** |

**Supplementary Figure S3. Enrichment analysis of the identified hits as KHK-C Inhibitors. (A)** ROC curve for the virtual screening performance of the identified hits as KHK-C inhibitors, demonstrating a perfect ROC score of 1.0. **(B)** Percentage of screened actives, showing that 100% of the actives appeared within the top 20% of the ranked results, indicating excellent enrichment.

**Supplementary
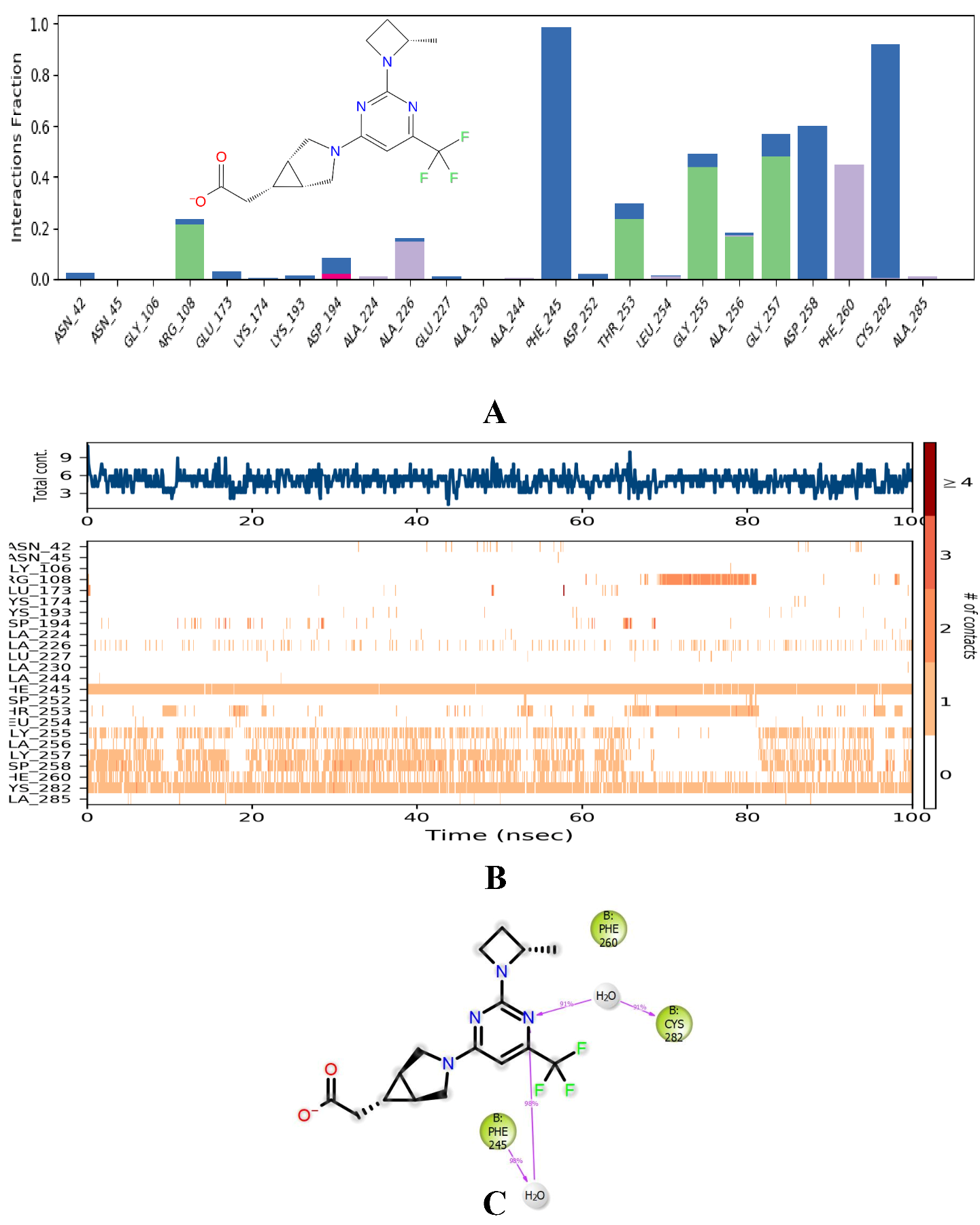
Figure S4. Post-MD simulations inferred results showing the protein-ligand interactions (A) Histogram illustrating various interaction of the reference ligand with KHK-C binding site residues. (B) Timeline representation of interactions. (C) 2D schematic summary of the interactions occurring over 30% of MD simulation run.**

**
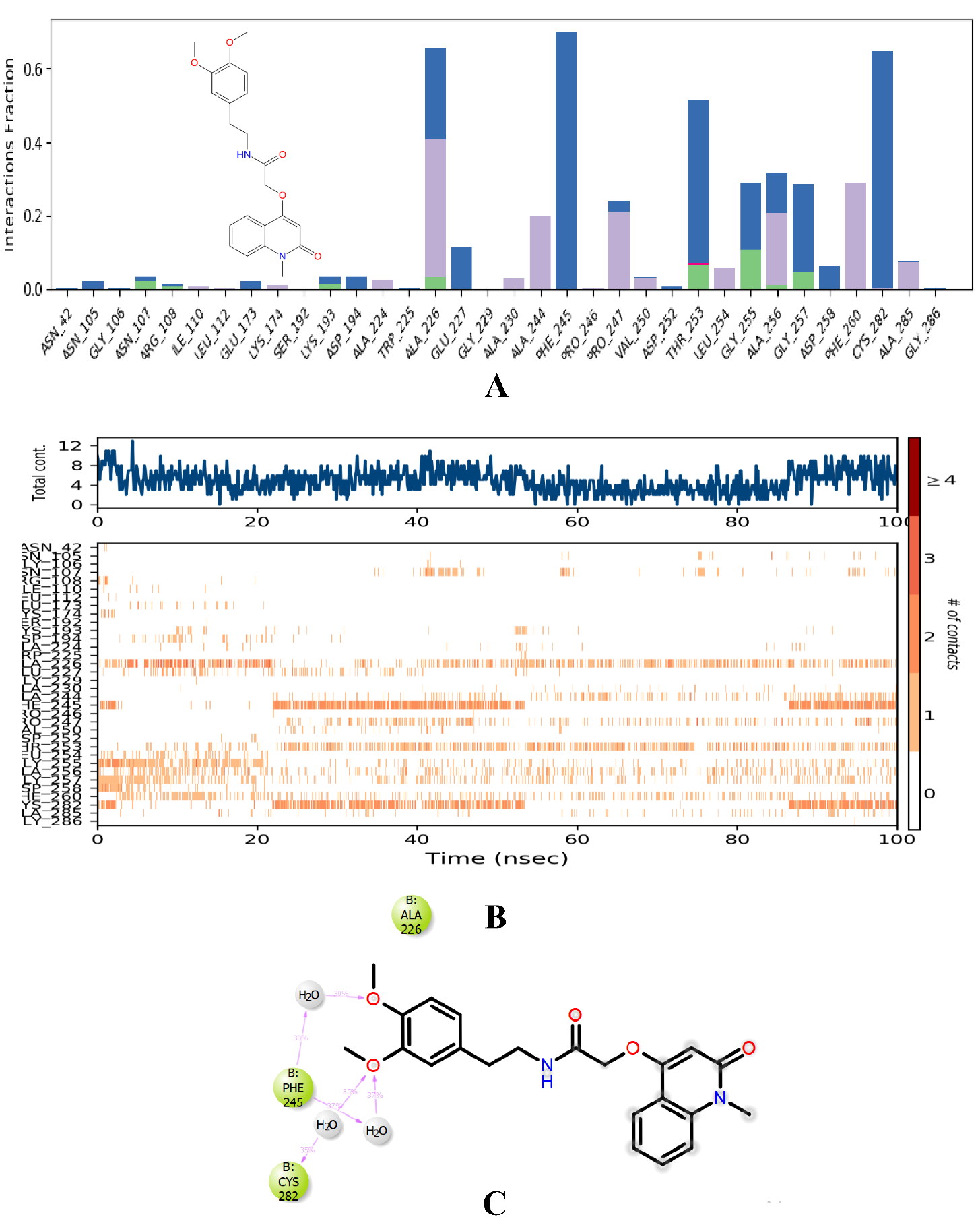
Supplementary Figure S5. Post-MD simulations inferred results showing the protein-ligand interactions (A) illustrating various interaction of hit 1 with KHK-C binding site residues. (B) Timeline representation of interactions. (C) 2D schematic summary of the interactions occurring over 30% of MD simulation run.**


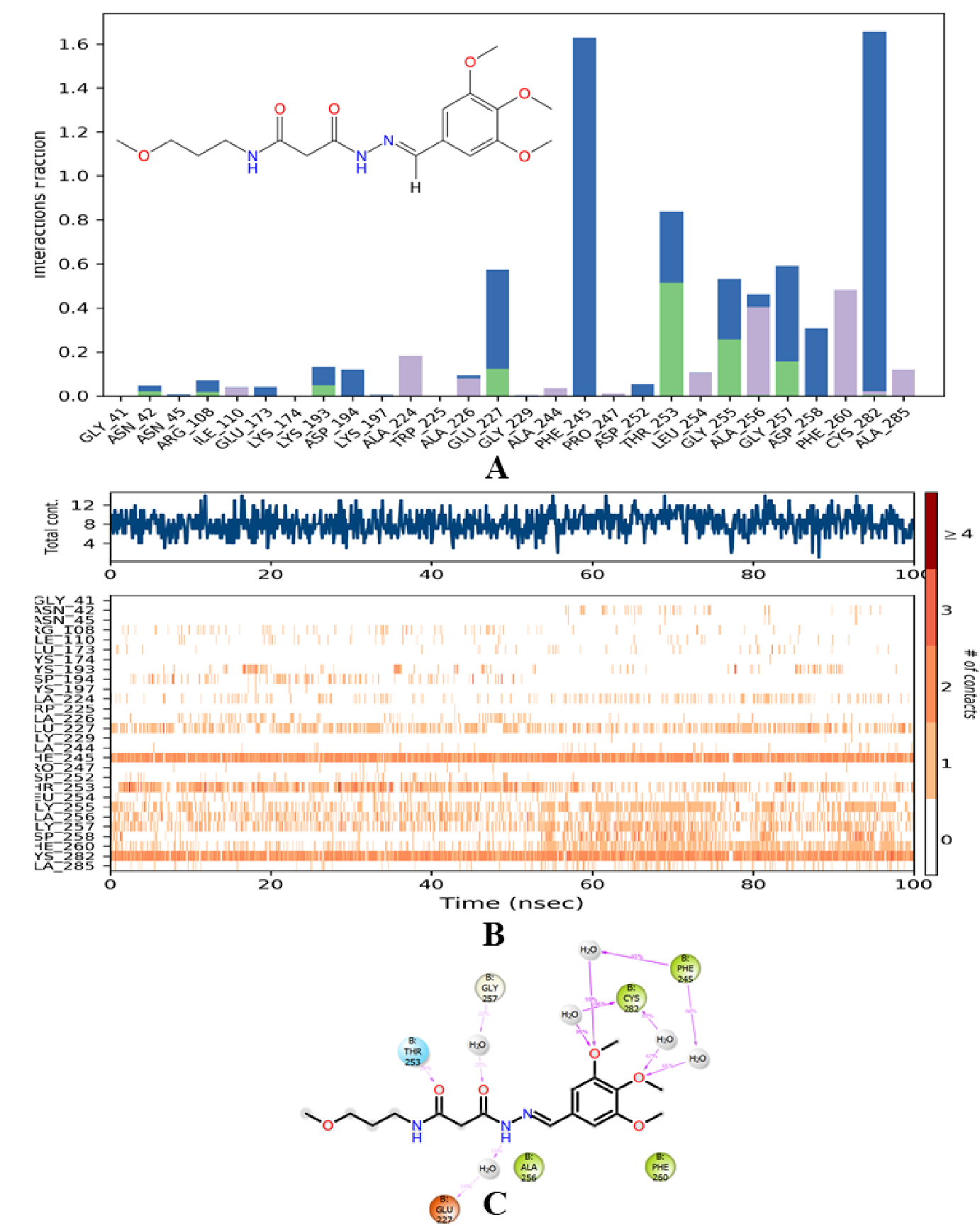


**Supplementary Figure S6. Post-MD simulations inferred results showing the protein-ligand interactions (A) Histogram illustrating various interaction of hit 2 with KHK-C binding site residues. (B) Timeline representation of interactions. (C) 2D schematic summary of the interactions occurring over 30% of MD simulation run.**

**
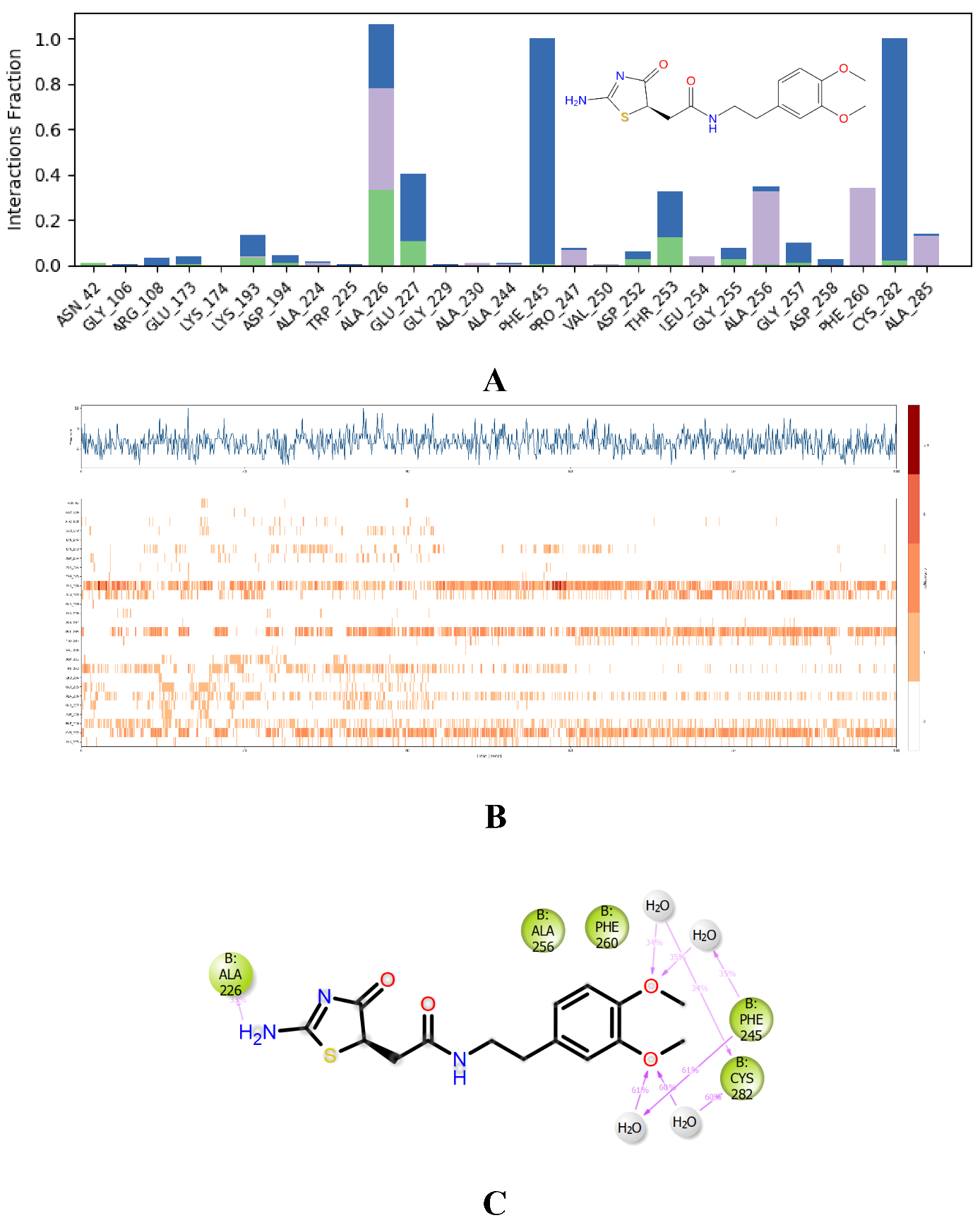
**

**Supplementary Figure S7. Post-MD simulations inferred results showing the protein-ligand interactions (A) Histogram illustrating various interaction of hit 4 with KHK-C binding site residues. (B) Timeline representation of interactions. (C) 2D schematic summary of the interactions occurring over 30% of MD simulation run.**

**
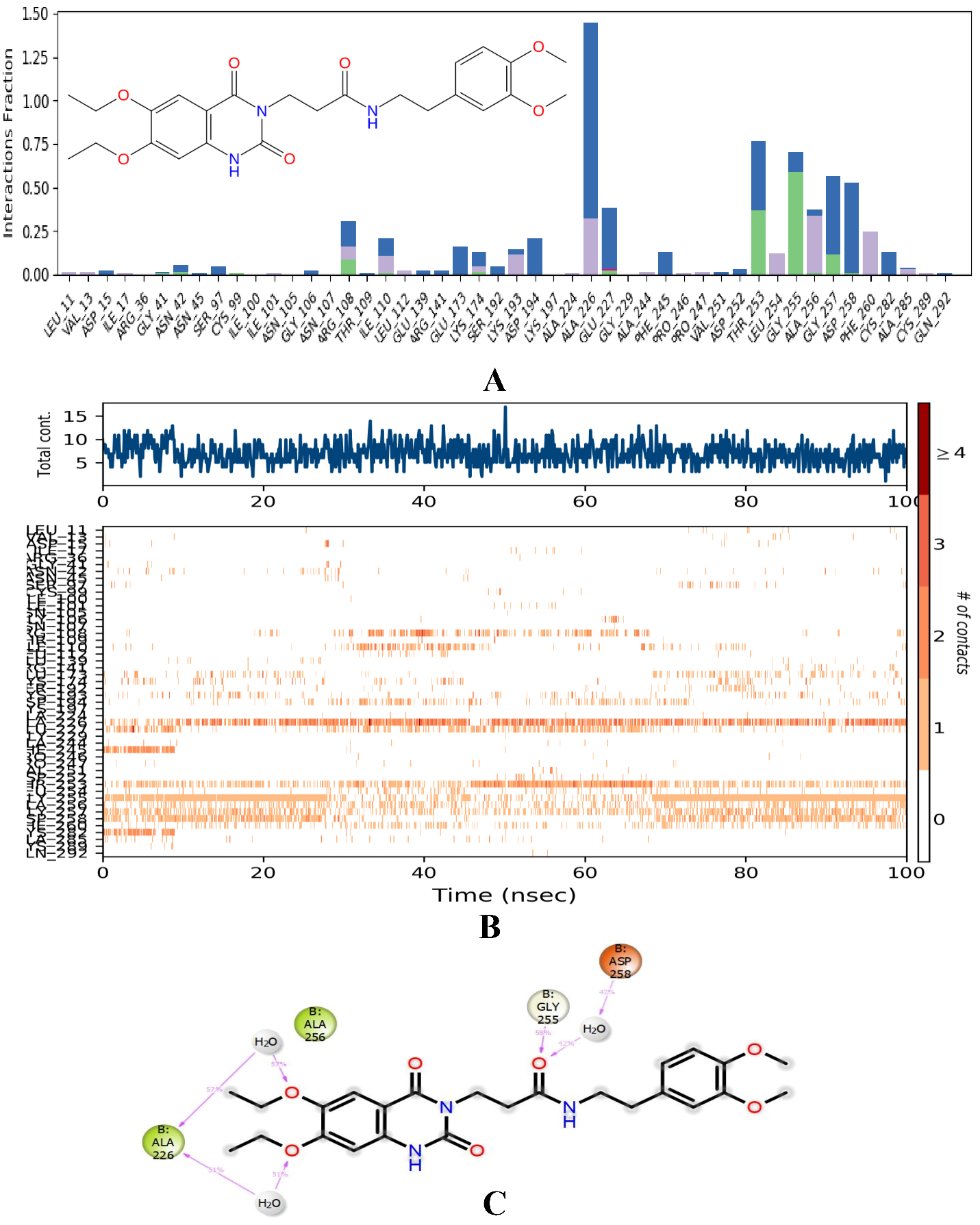
Supplementary Figure S8. Post-MD simulations inferred results showing the protein-ligand interactions (A) Histogram illustrating various interaction of hit 5 with KHK-C binding site residues. (B) Timeline representation of interactions. (C) 2D schematic summary of the interactions occurring over 30% of MD simulation run.**

**
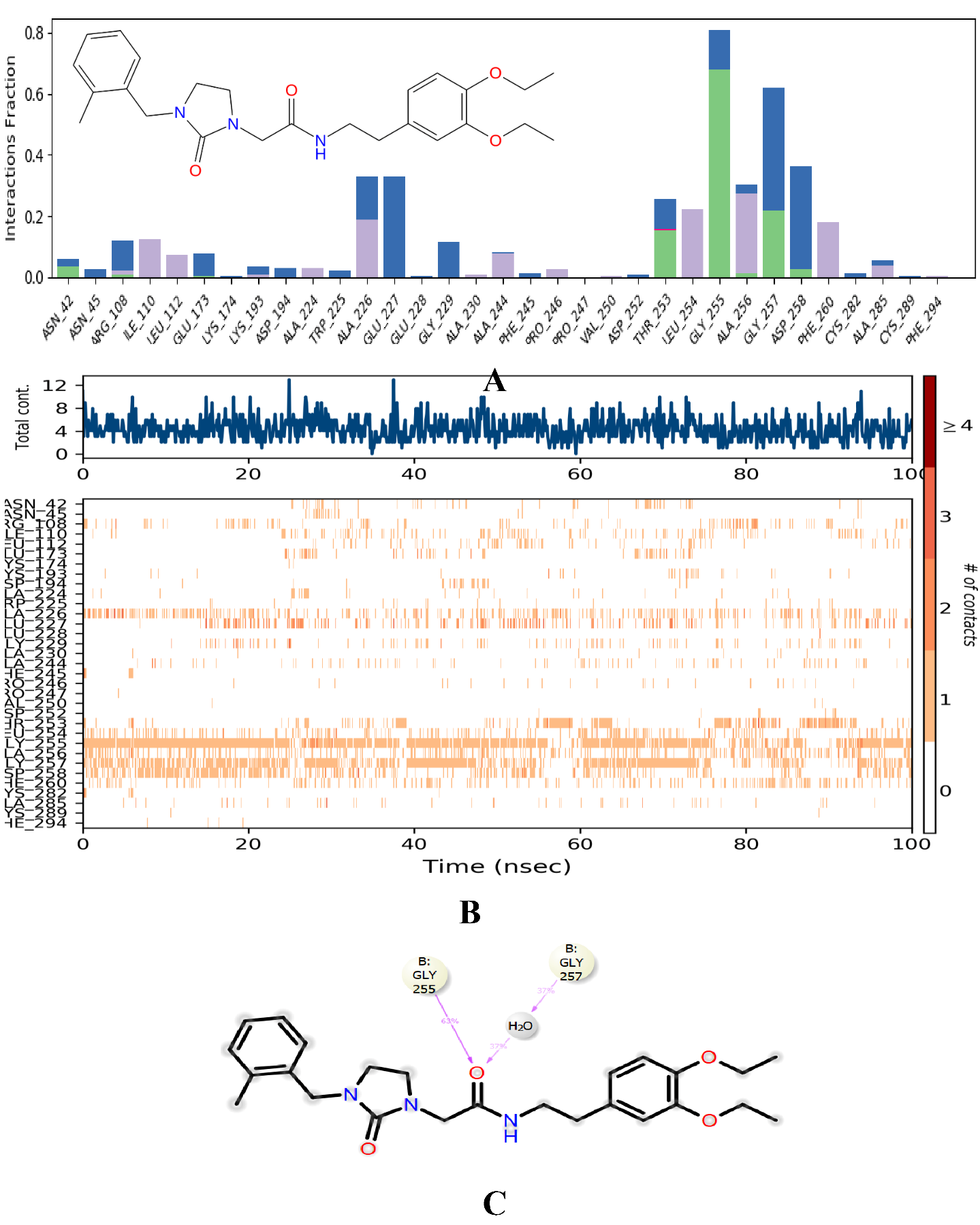
Supplementary Figure S9. Post-MD simulations inferred results showing the protein-ligand interactions (A) Histogram illustrating various interaction of hit 6 with KHK-C binding site residues. (B) Timeline representation of interactions. (C) 2D schematic summary of the interactions occurring over 30% of MD simulation run.**
